# Highly Regioselective Protecting-Group-Free Synthesis of the Antimalarial Drug MMV693183

**DOI:** 10.1021/acs.oprd.3c00353

**Published:** 2023-12-09

**Authors:** Pankaj V. Khairnar, Sarah L. Aleshire, Ravi Kumar Ongolu, Limei Jin, Michael G. Laidlaw, Kai O. Donsbach, B. Frank Gupton, Ryan C. Nelson, Charles S. Shanahan

**Affiliations:** Medicines for All Institute, Virginia Commonwealth University, Richmond, Virginia 23219, United States

**Keywords:** MMV693183, antimalarial, regioselective synthesis, protecting group free, API

## Abstract

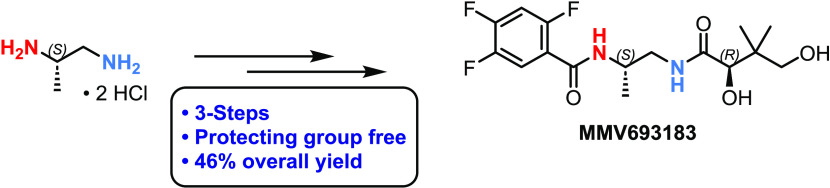

MMV693183 is a promising antimalarial drug candidate that works for uncomplicated malaria treatment and resistance management. Herein, we report an efficient and highly regioselective synthesis of MMV693183. This novel synthetic method highlights a three-step route with an overall yield of 46% from readily available starting materials. The key to the success lies in (1) utilizing the subtle difference of the two amino groups in the starting material (*S*)-propane-1,2-diamine dihydrochloride without amino protection and (2) identifying the *L*-(+)-tartaric acid as the counter acid for the organic salt formation, yielding the desired regioisomer up to 100:0. The efficient and scalable three-step protocol operates under mild conditions with a high chemo/regioselectivity, providing effective access to MMV693183.

## Introduction

**Figure 1:**
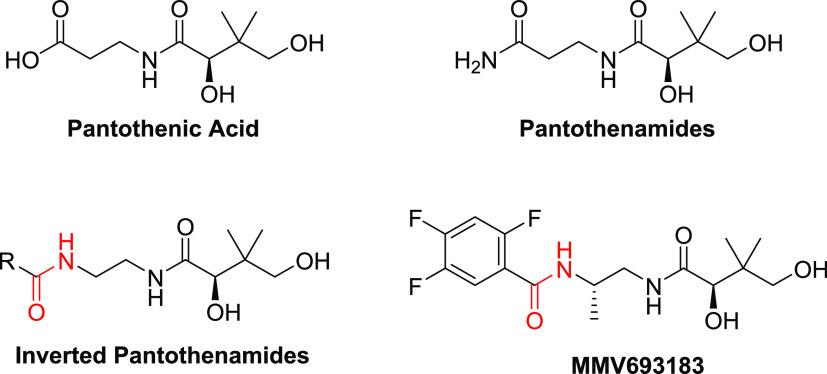
Chemical Structures of MMV693183 and related pantothenic acid derivatives.

Malaria remains one of the most devastating parasitic diseases, causing more than 241 million cases and 627 thousand estimated malaria deaths in 2020 according to the World Health Organization (WHO).^1^ The resistance to current antimalarial drugs and the high costs of treatment demand the search for new therapeutic agents.^2−5^ Pantothenic acid (vitamin B5) (Figure 1) is an important precursor to the enzyme cofactor coenzyme A (CoA), on which the predominant pathogen for Malaria, *Plasmodium falciparum*, is dependent during the intraerythrocytic stage of its life cycle.^6^ In the last few decades, many analogues of pantothenic acid have been synthesized that hinder pantothenic acid utilization and thus block the parasite life cycle.^7^ However, due to the poor stability of these carboxylic acids in human serum, they are not suitable as clinical candidates.^8−10^ Recently, the focus has been shifted toward the synthesis of pantothenamide and inverted pantothenamide analogues as they are resistant to the amidase enzyme, thus increasing stability in human serum (Figure 1).^11−13^ These inverted amide-bond pantothenamides (in red, Figure 1) are one class of such analogues that possess antiplasmodial activity.^10,12,15−17^

**Scheme 1:**
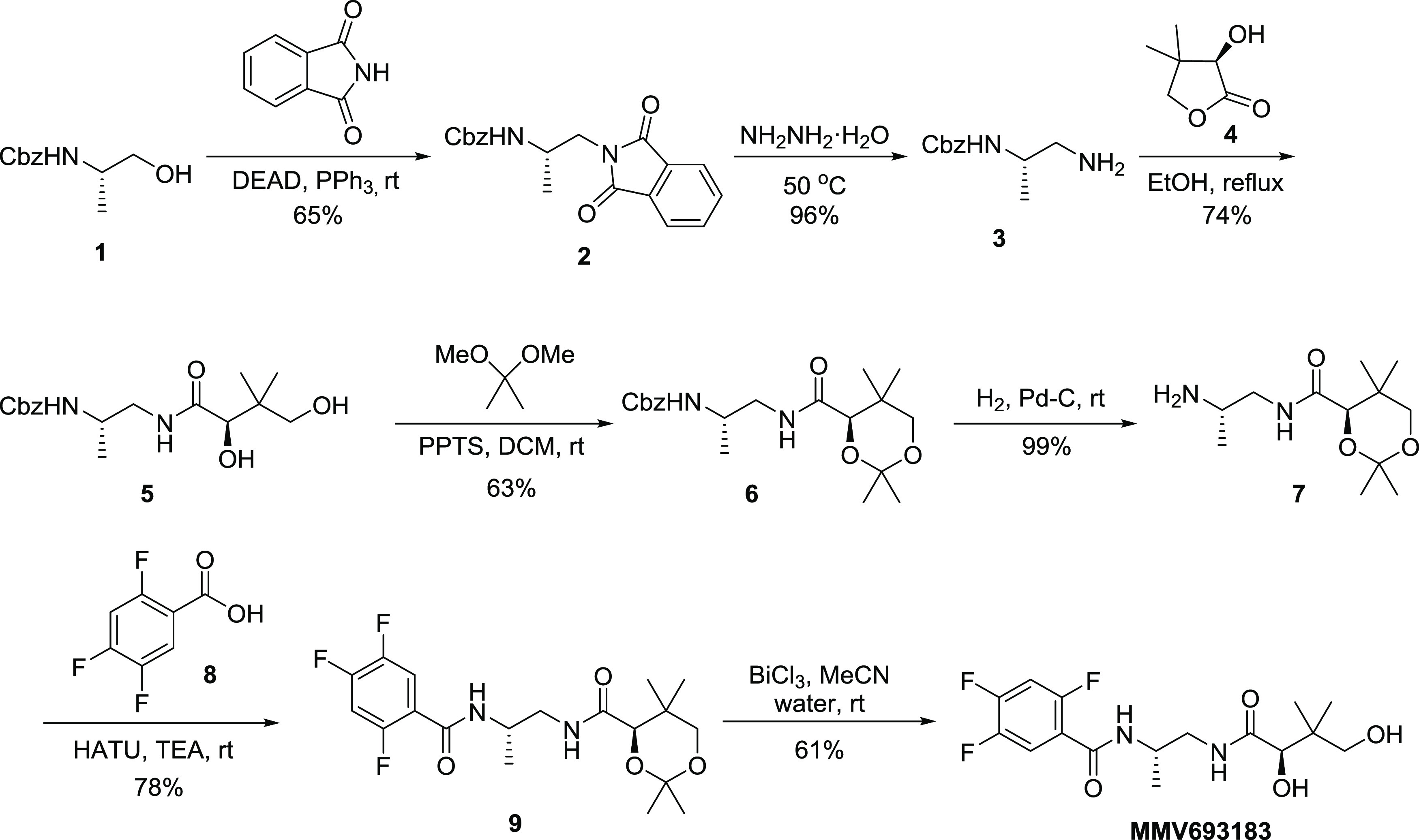
Reported Synthetic Route to MMV693183

Medicines for Malaria Venture (MMV) has developed the inverted pantothenamide MMV693183 (Figure 1) as a single dose treatment for uncomplicated malaria and resistance management. Developing a cost-effective process for the synthesis of MMV693183 will make the therapy more affordable and likely increase its impact. Unfortunately, only one synthetic route for MMV693183 has been published so far, and this route would be quite limiting to employ as a production route with a low price point in mind (Scheme 1).^14^ This route started with a Mitsunobu reaction of Cbz-protected aminoalcohol **1** with phthalimide to provide **2** in 65% yield, which was immediately subjected to phthalimide deprotection to provide the monoprotected diamine **3** in 96%. The resulting Cbz-protected diamine **3** was then reacted with (*R*)-pantolactone (**4**) to afford the diol **5** in 74% yield, which was then protected by 2,2-dimethoxypropane to provide acetonide **6** in 63% yield. Cbz deprotection by hydrogenolysis provided the free amine **7** in quantitative yield, which allowed for selective acylation of the amine with 2,4,5-trifluorobenzoic acid (**8**) to provide amide **9** in 78% yield. Finally, the acetonide protecting group was removed to furnish MMV693183 in 61% yield (14% overall yield).

While this 7-step sequence was successfully employed to make MMV693183 on a decagram scale, it also offers several opportunities for improvement. For example, the overall yield was only ∼14% and 3 of the 7 steps were used to manipulate protecting groups. The introduction of the less sterically hindered primary amine was accomplished via the Mitsunobu reaction and subsequent hydrazine deprotection, which are challenging transformations to scale-up due to their inherent wastefulness, cost, and the safety risks associated with handling diazodicarboxylates and hydrazine at scale. Thus, a more efficient and scalable route is needed for the synthesis of MMV693183 that would accommodate cost-effective commercial implementation and maximize access to this drug should it become commercially available.

## Results and Discussion

Herein, we report a three-step scalable synthesis of MMV693183 using readily available and low-cost starting materials, which avoids the use of any protecting group.^18^ Our approach is based on the hypothesis that the steric differences of both primary amines in (*S*)-1,2-diaminopropane dihydrochloride (**10**) would alone be sufficient to direct acylation to the desired less hindered amine (in blue) in a regioselective fashion (Table 1). To test this hypothesis, diamine **10** (readily available by resolution of the corresponding racemic diamine)^19−21^ was reacted directly with (*R*)-pantolactone (**4**) in the presence of 3 equiv of base (Table 1, entry 1). In this initial reaction, we observed good reactivity of the starting diamine; however, the mixture of amide products formed in the reaction was difficult to quantify and characterize.

**Table 1:**
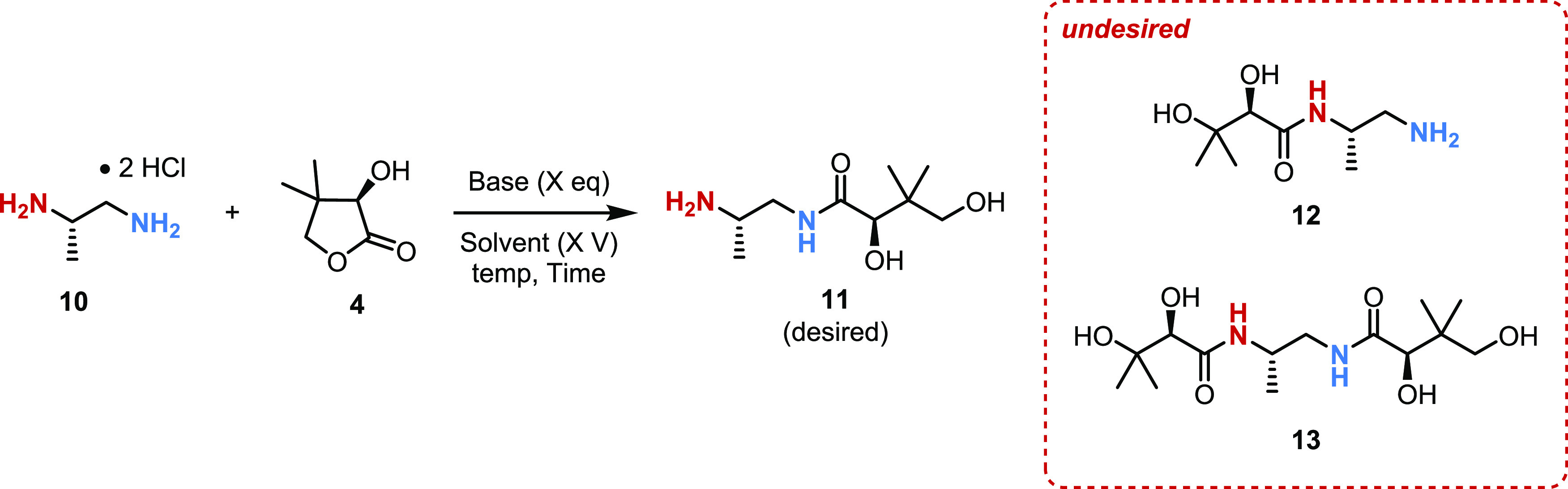
Direct Amidation of Diamine with (*R*)-Pantalactone^a^

					HPLC (area %)^b^		HILIC (area % ratio)^c^
entry	solvent	volume (V)	base	*T* (h)	13	11 + 12	11:12
1	EtOH	10	Na_2_CO_3_	3	8	52	89:11
2	MeOH	10	Na_2_CO_3_	3	4	82	90:10
3	IPA	10	Na_2_CO_3_	72	8	41	90:10
4	THF	10	Na_2_CO_3_	3	--e	--	--
5	CH_3_CN	10	Na_2_CO_3_	3	--e	--	--
6	DMF	10	Na_2_CO_3_	3	--e	--	--
7	DMSO	10	Na_2_CO_3_	3	--e	--	--
8	IPA/H_2_O (7:3)^d^	10	Na_2_CO_3_	3	5	86	91:09
9	IPA/H_2_O (7:3)	5	Na_2_CO_3_	3	7	84	90:10
10	IPA/H_2_O (7:3)	20	Na_2_CO_3_	3	6	84	92:08
11	IPA/H_2_O (7:3)	10	Et_3_N	3	6	88	91:09
12	IPA/H_2_O (7:3)	10	NaHCO_3_	3	--e	--	--
13	IPA/H_2_O (7:3)	10	NaOCH_3_	6	45	55	90:10
**14**	**IPA/H**_**2**_**O (9:1)**	**10**	**Na**_**2**_**CO**_**3**_	**3**	**4**	**86**	**90:10**
15^f^	IPA/H_2_O (9:1)	10	Na_2_CO_3_	6	11	86	90:10
16^g^	IPA/H_2_O (9:1)	10	Na_2_CO_3_	6	5	81	90:10
17^h^	IPA/H_2_O (9:1)	10	Na_2_CO_3_	6	6	83	90:10

a All reactions were carried out with (*S*)-propane-1,2-diamine dihydrochloride **10** (1.0 g 1.0 equiv), (*R*)-3-hydroxy-4,4-dimethyldihydrofuran-2(3*H*)-one **4** (1.0 equiv), base (3,0 equiv), 25 °C, 10 V of solvent, unless otherwise stated.

b LCAP at 210 nm.

c HILIC ratio at 210 nm.

d IPA: *i*-PrOH.

e No reaction.

f Reaction was carried out at 0 °C.

g (2.0 equiv) of Na_2_CO_3_ was used.

h (2.5 equiv) of Na_2_CO_3_ was used.

**Scheme 2:**
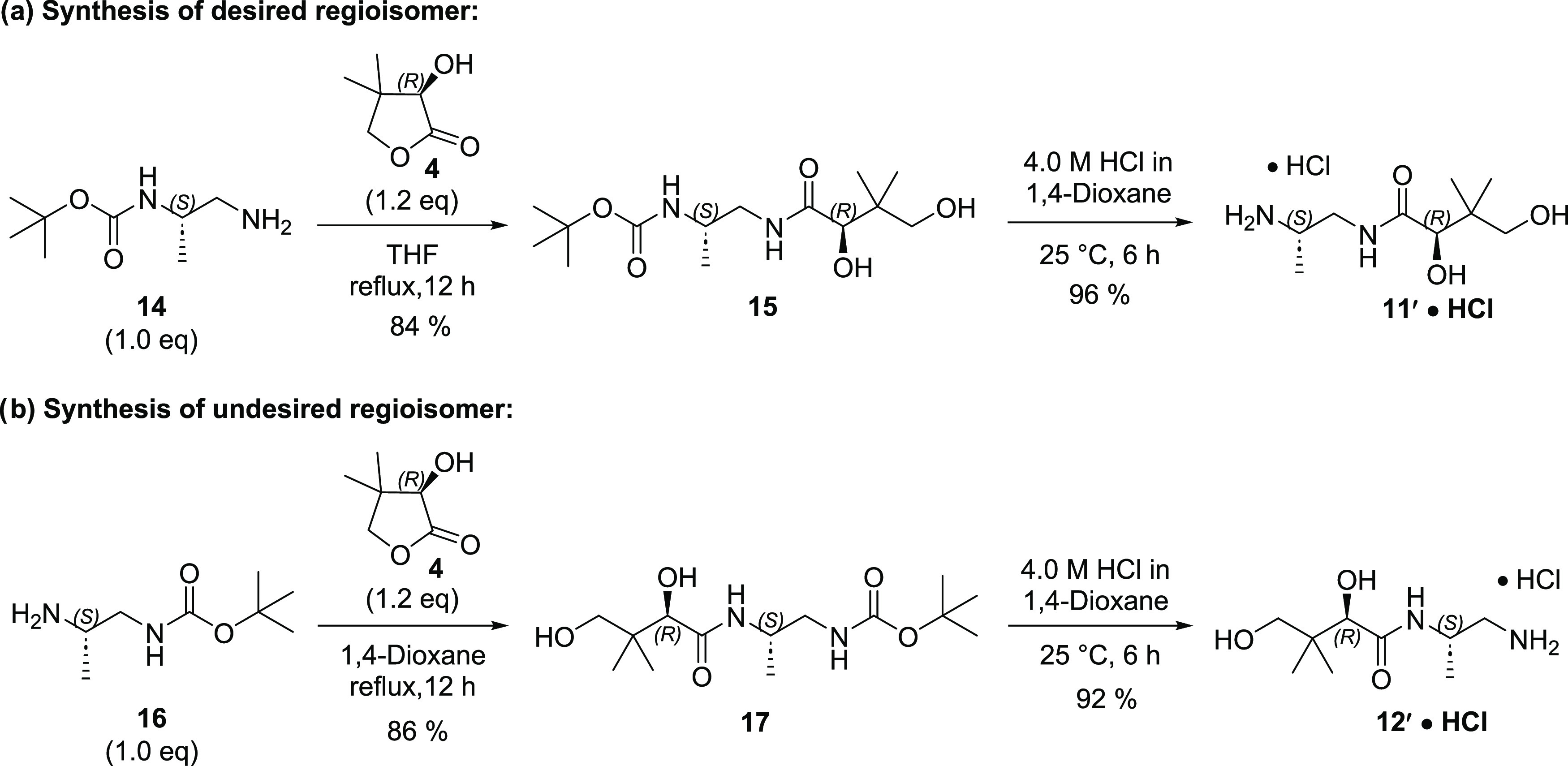
Synthesis of Regioisomers for HPLC Standards

In order to deconvolute the reaction mixture, we next discretely synthesized compounds **11′** and **12′** to utilize as analytical standards (Scheme 2). These were prepared by the reaction of (*R*)-pantolactone (**4**) with both Boc-protected amines **14** and **16** followed by Boc deprotection to provide pure **11′** and **12′** as their HCl salts.

Having the standards in hand allowed for the development of an HPLC method for the identification of the ratio of products in each direct amidation reaction (Table 1). A reversed-phase HPLC method was developed that was capable of separating the diamide products **13** from monoamide products **11′** and **12′**, but it could not competently separate the regioisomers **11′** and **12′**. A separate hydrophilic interaction liquid chromatography (HILIC) method, however, was able to separate regioisomers **11′** and **12′**, so a combination of HPLC and HILIC methods was used to characterize the reaction mixtures formed.

The initial reaction (Table 1, entry 1) with 3.0 equiv of Na_2_CO_3_ provided 52% assay yield (by HPLC area %) of a 9:1 regioisomeric mixture of monoamide products (**11** and **12**) favoring the desired product. It was also observed that 8% of the diacylated side product **13** formed in this reaction. For further optimization, a systematic solvent screening was conducted utilizing Na_2_CO_3_ as the base (Table 1, entries 2–8). It was determined that the reaction occurred only in polar protic solvents, such as MeOH, EtOH, and *i*-PrOH (Table 1, entries 1–3), but no reaction was observed in polar aprotic solvents probably due to insolubility of the inorganic base (Table 1, entries 4–7). In *i*-PrOH, the reaction was significantly slower, however, the addition of water to the solvent system (entries 9–15) gave superior results and up to 86% assay yield (by HPLC area %) of the monoamide products (Table 1, entry 8). Notably, the regioisomeric ratio was consistently 9:1 in favor of the desired isomer, regardless of the conditions screened. Due to the incomplete solubility of all species, 10 V of solvent was necessary to ensure proper mixing of the reaction mass as the reaction mixture in 5 V conditions (Table 1, entry 9) was difficult to stir. More dilute conditions (20 V of solvent) gave the same results as 10 V of the solvent (Table 1, Entry 10), so 10 V was deemed ideal.

With this optimized solvent system in hand, various bases were screened (Table 1, entries 11–13). Among these different bases, Na_2_CO_3_ and Et_3_N offered the best results; however, Et_3_N proved difficult in the workup, presumably due to the formation of the Et_3_N hydrochloride salt. Notably, the mixed solvents of *i*-PrOH/H_2_O (7:3) worked well; however, the product isolated by column chromatography from this mixed solvent system was of relatively low purity. The product was contaminated with inorganic salts, likely due to the high water content in the reaction mixture leading to isolation of the water-soluble product mixture along with significant amounts of dissolved inorganic salts. Decreasing the amount of water in the reaction from 7:3 to 9:1 (*i*-PrOH/H_2_O) allowed for effective isolation of the product with a higher isolated yield and purity (**11** + **12** 86% yield and >95%, qNMR purity) (Table 1, entry 14). Ultimately, the optimal conditions for this reaction (Table 1, entry 14) provided the mixture (∼9:1) of monoamide products **11** and **12** in 86% yield after column chromatography. The mixture of monoamides (**11** and **12**) was then taken to the next step for further separation.

With conditions to provide predominately the desired amide product **11**, the separation of the mixture to exclude the undesired secondary amide **12** (Table 2) was studied. The free amines are oily after chromatography, so, for depletion of the undesired isomer **12**, an acidic partner was sought that would generate a crystalline material. Thus, the 9:1 mixture of **11** and **12** was treated with various acids to screen for a suitable crystalline adduct. Mineral acids tend to cleave the amide bonds before crystallization occurs (Table 2, entries 1–3).

**Table 2:**
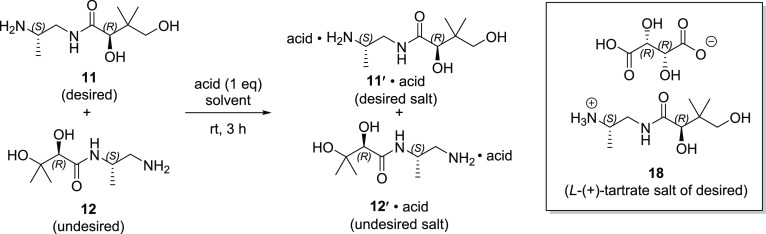
Purification of 11:12 Monoamide Mixture^a^

				HILIC (A % ratio)^b^
entry	acid	solvent	result	11′:12′
1	4.0 M HCl	1,4-dioxane	amide cleaved	ND
2	1.2 M HCl	IPA	amide cleaved	ND
3	H_2_SO_4_	EtOH	amide cleaved	ND
4	H_3_PO_4_	EtOH	no precipitate	NA
5	formic acid	EtOH	no precipitate	NA
**6**	benzoic acid	EtOH	no precipitate	NA
7	citric acid	EtOH	no precipitate	NA
8	d-(−)-tartaric acid	EtOH	no precipitate	NA
9^c^	**l-(+)-tartaric acid**	EtOH	**stable white salt**	25:1
10	l-(+)-tartaric acid	Methanol	no precipitate	NA
11	l-(+)-tartaric acid	Acetone	no precipitate	NA
12	l-(+)-tartaric acid	EtOAc	no precipitate	NA
13^d^	l-(+)-tartaric acid	*i*-PrOH	hygroscopic white salt	98:2
14^d^	l-(+)-tartaric acid	***i*****-PrOH/MeOH** (9:1)	**stable white salt**	100:0

a All of the reactions were carried out with ∼9:1 regioisomeric mixture of **11**:**12** (1.0 g, 1.0 equiv), acid (1.0 equiv), solvent (10 V), rt, 3h.

b HILIC ratio at 210 nm. ND = Not determined. NA = Not applicable.

c 80% of mass recovery.

d 78% of isolated yield.

With weaker H_3_PO_4_ or organic acids, the amide bond proved stable; however, most resulting salts that formed were not crystalline making them unsuitable for further purification (Table 2, entries 4–8). Intriguingly, when l-(+)-tartaric acid was utilized, a stable white solid (**18**) was formed, and more importantly, the precipitate was enriched to a 25:1 ratio of desired/undesired amides in ∼80% mass recovery and 73 area % (210 nm) purity (Table 2, entry 9). A variety of solvents screened for salt formation with l-(+)-tartaric acid, and it was found that cosolvents of *i*-PrOH/MeOH (9:1) gave a stable tartrate salt of the desired regioisomer (Table 2, entry 14), while other solvent systems were less effective. The salt formation process rejected the undesired regioisomer, effectively affording exclusively the desired regioisomer **18** along with some excess l-(+)-tartaric acid in the isolate, which created purification problems in the following step. With a substoichiometric loading of the l-(+)-tartaric acid (0.9 equiv), the desired amine salt **18** was isolated with 78% yield and 97 area % (210 nm) purity as the exclusive regioisomer.

**Scheme 3:**
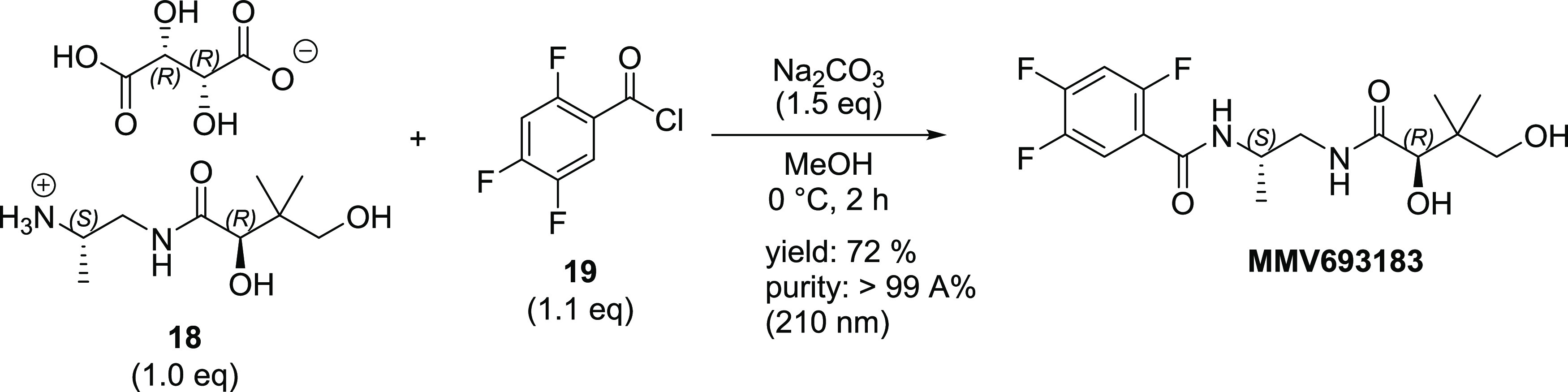
Completion of MMV693183 Synthesis

To complete the synthesis of MMV693183, l-(+)*-*tartrate salt **18** was reacted with 2,4,5-trifluorobenzoyl chloride (**19**) in the presence of 1.5 equiv of Na_2_CO_3_, providing the final API in 72% yield in >99 area % (210 nm) purity as determined by HPLC (Scheme 3). Both potassium and sodium carbonate can be used as a base to promote this final reaction, and both provide similar results; however, Na_2_CO_3_ is lower cost and was ultimately preferred.

**Scheme 4:**
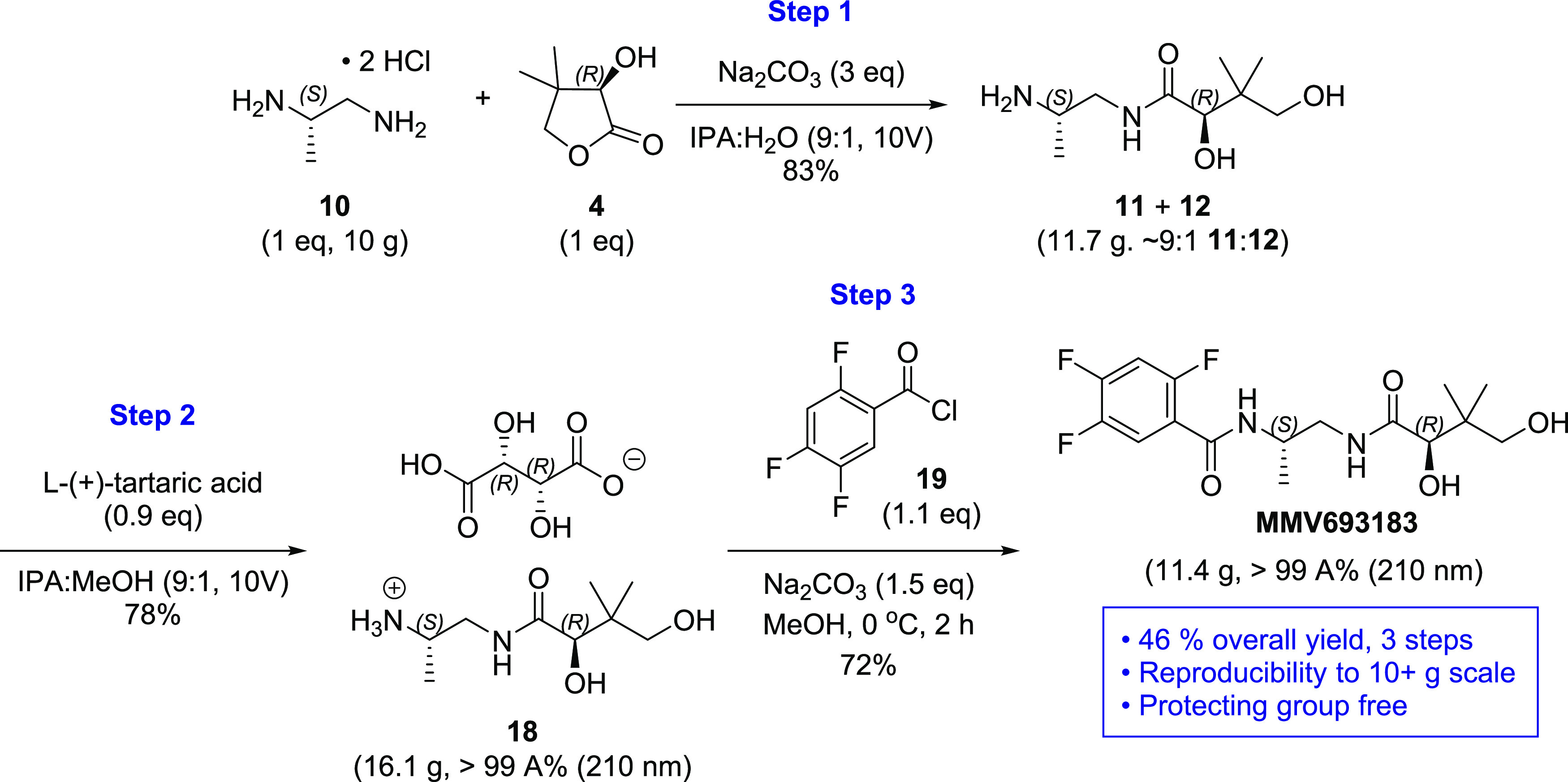
Gram-Scale Demonstration of Protecting-Group-Free Synthesis of MMV693183

To further showcase the synthetic utility of our three-step protocol for preparation of MMV693183, two multigram batches were carried out (Scheme 4). Starting with 10 g of (*S*)- 1,2-diaminopropane dihydrochloride (**10**), the monoamide product was isolated as a regioisomeric mixture (∼9:1) with 83% yield after column purification to remove the diamide impurity. Future work will explore the possibility to omit column chromatography. Treatment of the mixture of **11** and **12** with 0.9 equiv of l-(+)*-*tartaric acid furnished **18** in 78% yield with >99 area % (210 nm) HPLC purity. The resulting salt **18** was then acylated with 2,4,5-trifluorobenzoyl chloride (**19**) to afford MMV693183 in 72% yield with >99 area % (210 nm) HPLC purity. The overall yield of the three-step process from **10** to MMV693183 was 46%.

## Conclusions

Contrasting this newly developed synthetic route of MMV693183 with the prior reported route, a number of critical advantages have been achieved in this body of work, including (1) dramatically reducing the step count (3 vs 7) with a considerably higher overall yield (46 vs 14%); (2) eliminating the need for protecting groups by taking advantage of the native steric differences of the primary amines in **10**; (3) separating regioisomeric amides via an acid/base crystallization with l-(+)-tartaric acid; and (4) utilizing 2,4,5-trifluorobenzoyl chloride for acylation to avoid using expensive coupling reagents, all of this resulting in a cost-effective and scalable strategy to this promising API. These findings will hopefully serve to improve the commercial-scale manufacturing of MMV693183 in its effort to combat malaria.

## Experimental Section

### General Information

Reagents and solvents were purchased from Sigma-Aldrich Chemical Co., Fisher Scientific, Alfa Aesar, Acros Organics, Oakwood, or TCI. Liquid reagents were purified by distillation when necessary. Unless otherwise noted, solid reagents were used without further purification. The key starting materials, (*S*)-(−)-1,2-diaminopropane dihydrochloride and d-(−)-pantolactone, were purchased from Sigma-Aldrich with 98% purity and >99% ee, and they were used as is without further purification. Column chromatography was carried out using a Biotage Isolera automated flash chromatography system. Melting point was measured using the Stuart melting point apparatus SMP10. For all compounds, ^1^H, ^13^C, and ^19^F NMR spectra were recorded on a Bruker Avance III 600 MHz spectrometer. Chemical shifts were measured relative to the residual solvent resonance for ^1^H and ^13^C NMR (CDCl_3_ = 7.26 ppm for ^1^H and 77.2 ppm for ^13^C, DMSO-*d*_6_ = 2.50 ppm for ^1^H and 39.5 ppm for ^13^C, and CD_3_OD = 3.31 ppm for ^1^H and 49.0 ppm for ^13^C). Coupling constants *J* are reported in Hertz (Hz). The following abbreviations were used to designate signal multiplicity: s, singlet; d, doublet; t, triplet; dd, doublet of doublet; ddd, doublet of doublet of doublet; dt, double of triplet; m, multiplet; br, broad. Reactions were monitored by TLC, HPLC, or GC-MS by using various methods. Exact mass measurements were obtained on a Thermo Scientific LTQ Orbitrap Velos. Glassware was oven-dried at 120 °C, assembled while hot, and cooled to ambient temperature under an inert atmosphere. Unless noted otherwise, reactions involving air-sensitive reagents or requiring anhydrous conditions were performed under a nitrogen atmosphere. HRMS was recorded using PerkinElmer Axion 2 ToF MS, ionization mode: positive with scan range: 100–1000 *m*/*z*, flight tube voltage: 8 kV, spray voltage: 3.5 kV, and solvent: methanol.

#### Synthesis of (*R*)-*N*-((*S*)-2-aminopropyl)-2,4-dihydroxy-3,3-dimethylbutanamide (**11**)

Diamine hydrochloride **10** (10.0 g, 1.0 equiv, 68 mmol) and IPA/H_2_O (9:1, 100.0 mL, 10 V) was charged into a two-neck round-bottom flask, followed by the addition of Na_2_CO_3_ (21.6 g, 3.0 equiv, 204 mmol) at 25 °C. The reaction mixture was stirred at 25 °C for 2 h. The reaction mixture was cooled to 0 °C and lactone **4** (8.85 g, 1.0 equiv, 68 mmol) was added to this solution in one portion. The mixture was allowed to warm to 25 °C, and the reaction was monitored by HPLC. Once HPLC showed the complete conversion of lactone **4** (5 h), the mixture was diluted with methyl *t*-butyl ether (MTBE, 50 mL) to completely drive out the inorganic salts, and the solids were removed by filtration. The solid was washed with IPA (3 × 10 mL). The combined filtrate was evaporated to dryness under vacuum. The crude material was purified by column chromatography (gradient: DCM to 1:9 MeOH/DCM) to afford the monoamides of **11** and **12** (9:1 mixture, 11.7 g, 83%) as a colorless oil. ^1^H NMR (600 MHz, CD_3_OD) δ/ppm: 3.92 (s, 1H, major), 3.87 (s, 1H, minor), 3.48–3.41 (m, 2H, major and 2H, minor), 3.26–3.15 (m, 2H, major and 2H, minor), 3.14–3.05 (m, 1H, major and 1H, minor), 1.17 (d, *J* = 6.7 Hz, 3H, minor), 1.13 (d, *J* = 6.5 Hz, 3H, major), 0.99–0.91 (m, 6H, major and 6H, minor). ^13^C NMR (150 MHz, CD_3_OD) δ/ppm: 176.9 (major), 176.2 (minor), 77.7 (major), 71.5 (minor), 70.3 (major), 70.0 (minor), 48.3 (major), 48.0 (minor), 47.6 (minor), 47.0 (major), 40.7 (major), 40.3 (minor), 22.3 (minor), 21.9 (major), 21.5 (minor), 21.4 (major), 20.2 (major), 18.4 (minor). HRMS (ESI) *m*/*z*: [M + H]^+^ Calcd for C_9_H_21_N_2_O_3_: 205.1474; Found: 205.1437.

#### Synthesis of (*S*)-1-((*R*)-2,4-dihydroxy-3,3-dimethylbutanamido)propan-2-aminium Tartrate (**18**)

To a flask containing a mixture of **11** and **12** (9:1, 11.60 g, 1.0 equiv, 56.8 mmol) was charged with IPA/MeOH (9:1, 120 mL) followed by the addition of l-(+)-tartaric acid (7.67 g, 0.9 equiv, 51.1 mmol) at 25 °C. The reaction mixture was stirred at 25 °C overnight. The resulting white solid was filtered and washed with (3 × 20 mL) of IPA. The white solid was dried under a vacuum to give tartrate salt **18** (16.1 g, 78%, qNMR purity 99%). The tartrate salt was used for the next step without further purification. ^1^H NMR (600 MHz, DMSO-*d*_6_) δ/ppm: 8.12 (s, 1H), 6.31–4.76 (brs, 8H), 3.98 (s, 2H), 3.75 (s, 1H), 3.40–3.09 (m, 5H), 1.13 (d, *J* = 5.5 Hz, 3H), 0.83 (d, *J* = 7.0 Hz, 6H). ^13^C NMR (150 MHz, DMSO*-d*_6_) δ/ppm: 174.7, 174.1, 75.2, 72.04, 72.01, 67.7, 46.4, 41.7, 21.4, 20.6, 16.2. HRMS (ESI) *m*/*z*: [M + H]^+^ Calcd for C_9_H_21_N_2_O_3_: 205.1552; Found: 205.1539.

#### Synthesis of *N*-((*S*)-1-((*R*)-2,4-dihydroxy-3,3-dimethylbutanamido)propan-2-yl)-2,4,5-trifluorobenzamide (**MMV693183**)

To a vacuum-dried two-neck round-bottom flask were added tartrate salt **18** (14.67 g, 1.0 equiv) and dry MeOH (147.0 mL, 10 V) followed by the addition of Na_2_CO_3_ (8.82 g, 2.0 equiv) at 25 °C under nitrogen. The reaction mixture was stirred for 1h at the same temperature. After 1h, the reaction mixture was cooled to 0 °C and trifluorobenzoyl chloride **19** (5.86 mL, 1.1 equiv) was added dropwise. The mixture was stirred for another 2 h at 0 °C. After completion (monitored by HPLC), the reaction mixture was diluted with MTBE (70 mL) and the insoluble salts were filtered off, and the cake was washed with MeOH (3 × 10 mL). The combined organic layers were evaporated to dryness. The resulting crude mixture was purified by column chromatography (gradient: hexanes to 1:9 EtOAc/hexanes) to give the pure product as a white solid (11.4 g, 72%, > 99% HPLC A% purity at 210 nm). ^1^H NMR (600 MHz, DMSO-*d*_6_) δ/ppm: 8.24 (d, *J* = 7.5 Hz, 1H), 7.85 (t, *J* = 6.2 Hz, 1H), 7.74–7.60 (m, 2H), 5.42 (d, *J* = 5.4 Hz, 1H), 4.46 (t, *J* = 5.6 Hz, 1H), 4.09–3.98 (m, 1H), 3.73 (d, *J* = 5.4 Hz, 1H), 3.31–3.24 (m, 2H), 3.19–3.11 (m, 2H), 1.10 (d, *J* = 6.7 Hz, 3H), 0.76 (d, *J* = 6.0 Hz, 6H). ^13^C NMR (150 MHz, DMSO-*d*_6_) δ/ppm: 173.6, 161.3, 154.7 (ddd, *J* = 250.0, 10.0, 2.2 Hz), 150.2 (dt, *J* = 253.0, 14.4 Hz), 145.8 (ddd, *J* = 244.0, 12.8, 3.0 Hz), 121.1 (dt, *J* = 16.5, 4.4 Hz), 118.0 (dd, *J* = 20.1, 4.2 Hz), 106.9 (dd, *J* = 29.6, 8.0 Hz), 68.0, 45.9, 42.9, 39.0, 20.9, 20.2, 18.0. ^19^F NMR (564 MHz, DMSO-*d*_6_) δ/ppm: – 114.5 (dd, *J* = 16.1, 5.8 Hz, 1F), – 131.0 (dd, *J* = 23.1, 5.8 Hz, 1F), – 142.8 (dd, *J* = 24.5, 8.2 Hz, 1F). HRMS (ESI) *m*/*z*: [M + Na]^+^ Calcd for C_16_H_21_F_3_N_2_O_4_Na: 385.1351; Found: 385.1448. Melting point: 101 °C.

### Syntheses of MMV693183 Regioisomers from Corresponding HCl Salts **11′** and **12′**

#### Synthesis of *tert*-butyl ((*S*)-1-((*R*)-2,4-dihydroxy-3,3-dimethylbutanamido)propan-2-yl)carbamate (**15**)

To a mixture of N-Boc amine **14** (1.0 g, 1.0 equiv, 6 mmol) in dry THF (10 mL) was added lactone **4** (0.9 g, 1.2 equiv, 7 mmol) in one portion at 25 °C under a nitrogen atmosphere and the resulting mixture was refluxed for overnight in an oil bath. After the completion of the reaction (monitored by TLC), the reaction mixture was allowed to cool to 25 °C. The organic solvent was removed under vacuum, and the crude mixture was purified by column chromatography (gradient: hexanes to 1:9 EtOAc/hexanes) to obtain the pure desired regioisomer **15** (1.5 g, 89%). ^1^H NMR (600 MHz, DMSO-*d*_6_) δ/ppm: 7.75 (t, *J* = 5.6 Hz, 1H), 6.66 (d, *J* = 7.5 Hz, 1H), 5.39 (brs, 1H), 4.45 (brs, 1H), 3.37 (s, 1H), 3.59–3.50 (m, 1H), 3.30 (d, *J* = 10.4 Hz, 1H), 3.18 (d, *J* = 10.4 Hz, 1H), 3.16–3.10 (m, 1H), 3.05–2.98 (m, 1H), 1.37 (s, 9H), 0.98 (d, *J* = 6.5 Hz, 3H), 0.81 (s, 3H), 0.79 (s, 3H). ^13^C NMR (150 MHz, DMSO-*d*_6_) δ/ppm: 173.4, 155.0, 77.5, 75.1, 68.1, 46.2, 43.3, 38.9, 28.2, 20.9, 20.3, 18.5. HRMS (ESI) *m*/*z*: [M + Na]^+^ Calcd for C_14_H_28_N_2_O_5_Na: 327.1896; Found: 327.1881.

#### Synthesis of (*R*)-*N*-((*S*)-2-aminopropyl)-2,4-dihydroxy-3,3-dimethylbutanamide Hydrochloride (**11′**)

To a flask containing compound **15** (1 g, 1.0 equiv, 3.3 mmol) was added the 4.0 M HCl in dioxane (8.2 mL, 10.0 equiv, 33 mmol) at 25 °C and the resulting mixture was stirred for 5–6 h and between this time a white solid was precipitated. After the reaction was completed (monitored by TLC), the precipitates were collected by filtration. The filter cake was washed with cold EtOH to afford the desired pure hydrochloride salt **11′** (0.72 g, 91%). The HCl salt was very hygroscopic and was used for the next step without further purification. ^1^H NMR (600 MHz, CD_3_OD) δ/ppm: 4.16 (s, 1H), 3.99 (dd, *J* = 15.6, 6.7 Hz, 2H), 3.69–3.65 (m, 1H), 3.33–3.30 (m, 1H), 3.15 (dd, *J* = 13.0, 4.1 Hz, 1H), 1.44 (d, *J* = 6.9 Hz, 3H), 1.07 (s, 3H), 1.01 (s, 3H). HRMS (ESI) *m*/*z*: [M + H]^+^ Calcd for C_9_H_21_N_2_O_3_: 205.1552; Found: 205.1532.

#### Synthesis of *tert*-butyl ((*S*)-2-((*R*)-2,4-dihydroxy-3,3-dimethylbutanamido)propyl)carbamate (**17**)

To a mixture of N-Boc amine **16** (1 g, 1.0 equiv, 6 mmol) in dry 1,4-dioxane (10.0 mL) was added lactone **4** (0.9 g, 1.2 equiv, 7 mmol) in one portion under a nitrogen atmosphere at 25 °C and the resulting mixture was refluxing for 12 h using an oil bath. After the completion of the reaction (monitored by TLC), the reaction mixture was allowed to cool to 25 °C. The organic solvent was removed under vacuum, and the crude mixture was purified by column chromatography (gradient: hexanes to 1:9 EtOAc/hexanes) to obtain the pure undesired regioisomer **17** (1.46 g, 86%). ^1^H NMR (600 MHz, DMSO-*d*_6_) δ/ppm: 7.44 (d, *J* = 8.3 Hz, 1H), 6.73 (d, *J* = 5.6 Hz, 1H), 4.75 (brs, 2H), 3.89–3.83 (m, 1H), 3.69 (s, 1H), 3.28 (d, *J* = 10.4 Hz, 1H), 3.17 (d, *J* = 10.4 Hz, 1H), 3.00–2.90 (m, 2H), 1.35 (s, 9H), 0.99 (d, *J* = 6.7 Hz, 3H), 0.80 (s, 3H), 0.78 (s, 3H). ^13^C NMR (150 MHz, DMSO-*d*_6_) δ/ppm: 172.5, 155.8, 77.5, 75.1, 68.1, 44.9, 44.3, 39.0, 28.2, 21.0, 20.4, 17.8. HRMS (ESI) *m*/*z*: [M + Na]^+^ Calcd for C_14_H_28_N_2_O_5_Na: 327.1998; Found: 327.1993.

#### Synthesis of (*R*)-*N*-((*S*)-1-aminopropan-2-yl)-2,4-dihydroxy-3,3-dimethylbutanamide Hydrochloride (**12′**)

A mixture of compound **17** (1 g, 1.0 equiv, 3 mmol) and 4.0 M HCl in dioxane (8.0 mL, 10.0 equiv, 30 mmol) was stirred at 25 °C for 5–6 h, upon which a white solid was precipitated out. After the reaction was completed (monitored by TLC), the white solid was collected by filtration. The resulting filter cake was washed with cold EtOH to afford pure hydrochloride salt **12′** (0.68 g, 86%). The HCl salt was not stable and very hygroscopic. It was used for the next step without further purification. ^1^H NMR (600 MHz, CD_3_OD) δ/ppm: 4.28–4.15 (m, 1H), 3.78 (s, 1H), 3.63 (d, *J* = 11.4 Hz, 1H), 3.29 (d, *J* = 11.3 Hz, 1H), 3.07 (dd, *J* = 13.0, 4.1 Hz, 1H), 2.96 (dd, *J* = 13.1, 3.8 Hz, 1H), 1.26 (d, *J* = 7.0 Hz, 3H), 1.05 (s, 3H), 0.94 (s, 3H). HRMS (ESI) *m*/*z*: [M + H]^+^ Calcd for C_9_H_21_N_2_O_3_: 205.1474; Found: 205.1492.

## Supporting Information Available

Detailed HPLC-HILIC method development, copies of NMR spectra of all compounds, detailed synthetic procedure and analytical data of the undesired regioisomer of MMV693183 (PDF)
