# Supplementary material for: Highly Regioselective Protecting-Group-Free Synthesis of the Antimalarial Drug MMV693183
Source: Org Process Res Dev. 2023 Dec 9;28(1):273–80. doi: 10.1021/acs.oprd.3c00353 (PMC10804412; doi:10.1021/acs.oprd.3c00353)
Supplement: Supplementary file 1 — op3c00353_si_001.pdf [file op3c00353_si_001.pdf]

Highly Regioselective Protecting Group-Free Synthesis of the Antimalarial Drug MMV693183

Pankaj V. Khairnar, Sarah L. Aleshire, Ravi Kumar Ongolu, Limei Jin, Michael G. Laidlaw, Kai O. Donsbach, B. Frank Gupton, Ryan C. Nelson, Charles S. Shanahan**

Medicines for All Institute, Virginia Commonwealth University, Richmond, VA, 23219, USA

*Email: shanahanc@vcu.edu; rnelsonchem@gmail.com

Table S1. Analytical HPLC-HILIC Method for the separation of 1st step regioisomers

<table>
<tr><td colspan="3">Structures & IDs:</td></tr>
<tr><td colspan="3">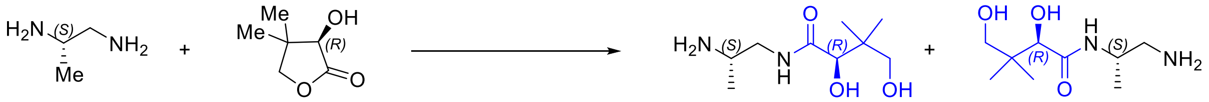

10 Diamine — 4 Lactone — 11 Desired amide — 12 Undesired amide</td></tr>
<tr><td colspan="3"><u>Conditions:</u></td></tr>
<tr><td colspan="3"><u>Column:</u> Agilent Poroshell 120 HILIC-Z (3.0 x 100 mm, 2.7 µm)</td></tr>
<tr><td colspan="3"><u>Mobile Phase A:</u> Acetonitrile</td></tr>
<tr><td colspan="3"><u>Mobile Phase B:</u> 25 mM ammonium formate adjusted to pH 3.0 ± 0.1 with formic acid</td></tr>
<tr><td><u>Injection volume:</u> 1 µL</td><td><u>Column temp:</u> 30 °C</td><td><u>Flow rate:</u> 1.0 mL/min</td></tr>
<tr><td colspan="3"><u>Detector wavelength(s):</u> 210 nm</td></tr>
<tr><td><u>LC Gradient Table:</u>
<table><tr><td>Time (min)</td><td>%A</td><td>%B</td></tr></table></td><td colspan="2"><u>Sample preparation:</u> Prepare samples at approximately 1.0 mg/mL in methanol</td></tr>
</table>

0.0	90%	10%
12.0	90%	10%

Post-run equilibration: N/A

Retention Times

Compound	Time (min)	Relative RF (mg/mL)*	Relative RF (M)*
Desired amide regioisomer **11**	6.6		
Undesired amide regioisomer **12**	7.4		

<u>**Notes:**</u>

- Ensure thorough equilibration (thermal and mobile phase) prior to starting analyses
- $*Relative\ RF = \frac{\left(Analyte\ 2\ Conc./_{Analyte\ 2\ Peak\ Area}\right)}{\left(Analyte\ 1\ Conc./_{Analyte\ 2\ Peak\ Area}\right)}$

Representative Chromatogram(s) (attach additional chromatograms and spectra as needed)

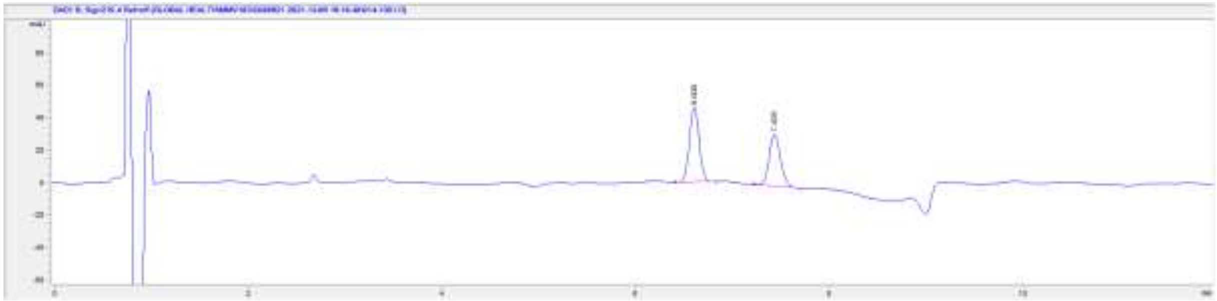

Desired amide 11:

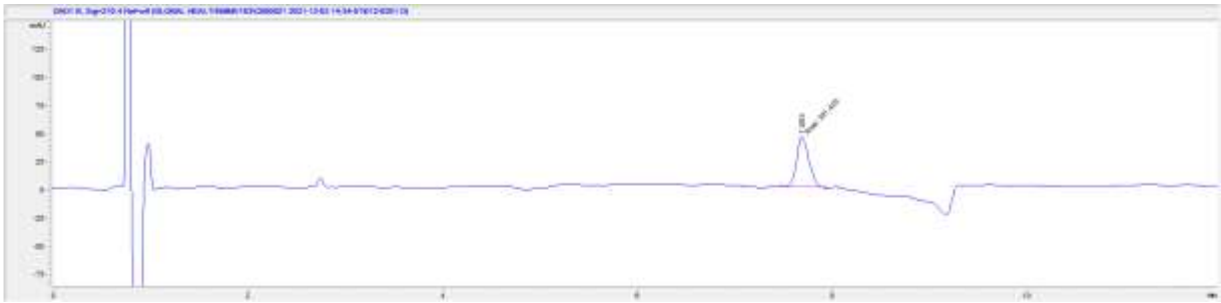

Undesired amide 12:

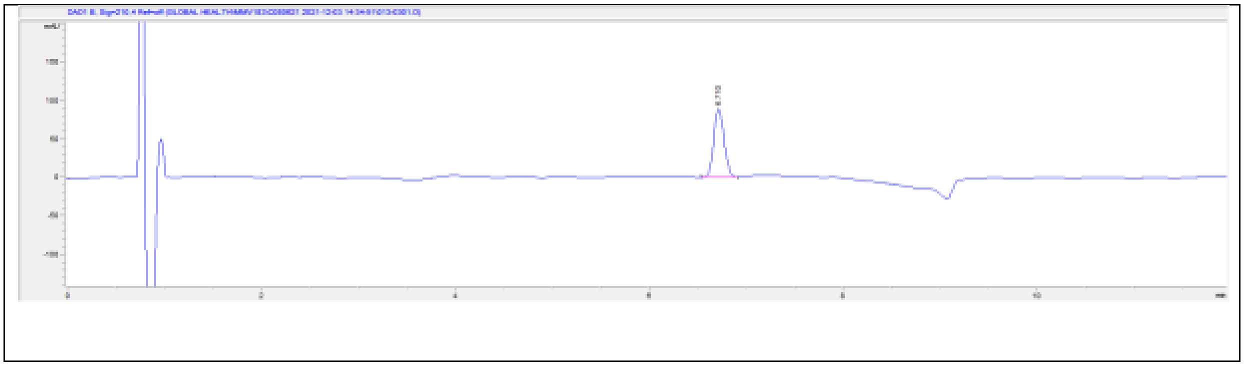

Scheme S1. Step-II Pictures of Tartrate Salt Formation (18)

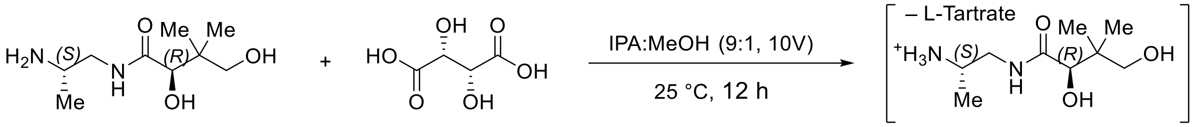

9:1 Mix (**11:12**)
1.0 equiv
Column Purified material

L-(+)Tartaric acid
0.9 equiv

18

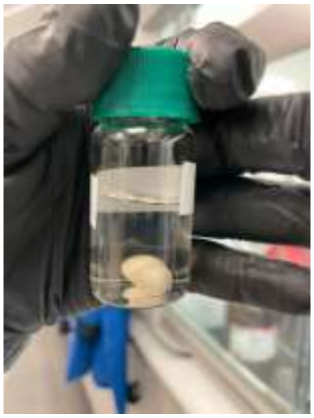

9:1 mix of regioisomers in 10 V of IPA:MeOH.

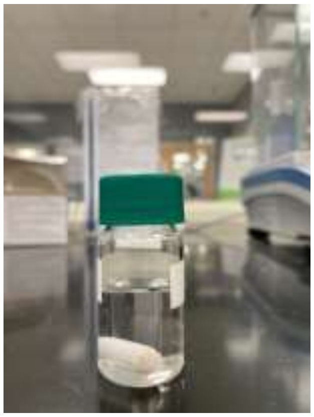

Immediately after addition of 0.9 eq. of *L-(+)*-tartaric acid

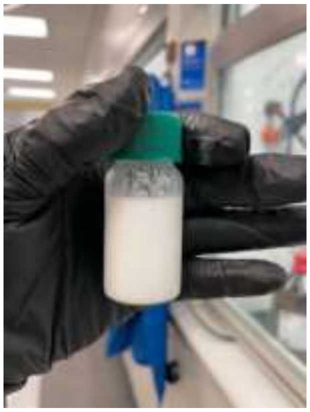

After 12 h, a white solid precipitates out.

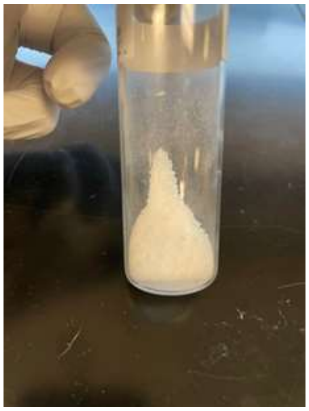

Isolated pure desired tartrate salt after filtration and drying.

Scheme S2. Step-III Pictures of *N*-Acylation Reaction

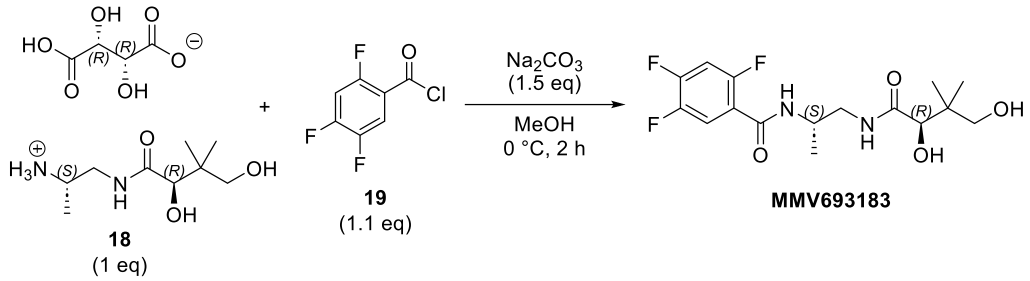

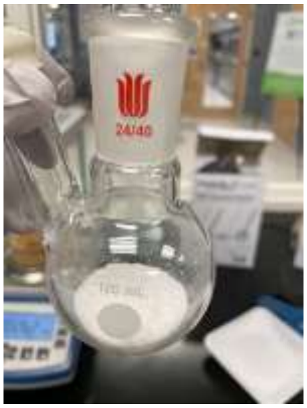

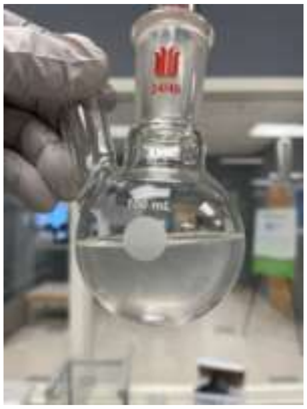

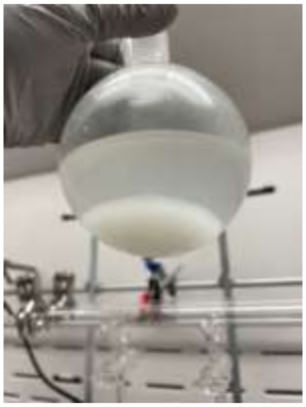

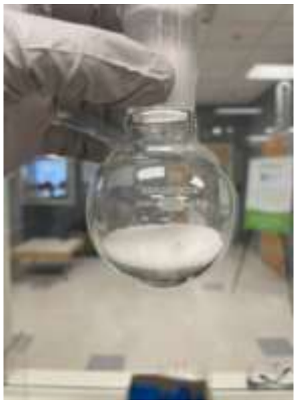

Pure Desired Tartrate salt **18**	Immediately after addition of 10 V of dry MeOH.	After stirring with Na_2CO_3 at 0 °C for 2h, salts precipitate out.	Isolated Desired-MMV693183 after column purification.

Table S2. Analytical HPLC Method for the separation of 3rd step regioisomers

This HPLC method did not resolve the desired and undesired regioisomers of 1st step.

Structures & IDs:

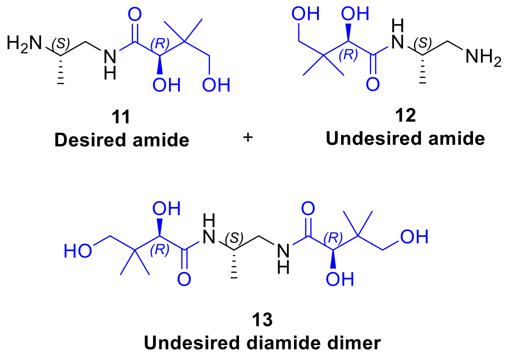

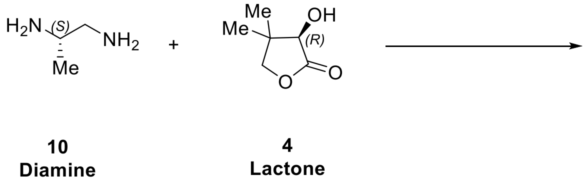

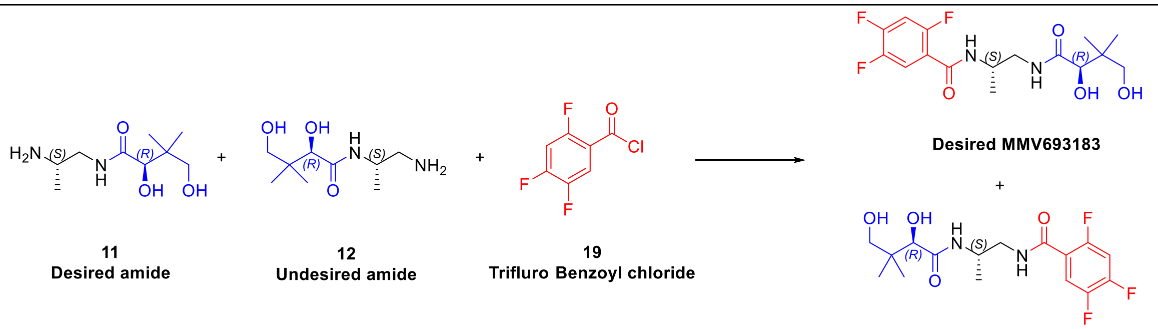

11 Desired amide

12 Undesired amide

19 Trifluro Benzoyl chloride

Desired MMV693183

Undesired MMV693183

<u>Conditions:</u>

<u>Column:</u> Agilent Eclipse XDB-C18 (4.6 x 250 mm, 5 µm)

<u>Mobile Phase A:</u> 0.1% phosphoric acid in water (1 mL HPLC grade H_3PO_4 in 1000 mL HPLC grade water)

<u>Mobile Phase B:</u> Methanol

<u>Injection volume:</u> 1 µL | <u>Column temp:</u> 40 °C | <u>Flow rate:</u> 1.5 mL/min

<u>Detector wavelength(s):</u> 210 nm

<u>LC Gradient Table:</u>

Time (min)	%A	%B
0.0	90%	10%
0.5	90%	10%
5.0	55%	45%
14.0	55%	45%

Post-run equilibration: 4 min

<u>Sample preparation:</u> Prepare samples at approximately 1.0 mg/mL in ethanol. Step two samples will require sonication to help convert the trifluoro benzoyl chloride to its ethyl ester for monitoring.

Retention Times

**Compound**	**Time (min)**	**Relative RF (mg/mL)***	**Rlative RF (M)***
Desired amide regioisomer **11**	2.3		
Undesired amide regioisomer **12**	2.3		
Lactone **4**	4.5		
Diamide dimer **13**	6.6		
Trifluoro benzoyl chloride (as ethyl ester) **19**	10.1		

**Undesired MMV-183** regioisomer	11.5		
**Desired MMV-183** regioisomer	11.8		

Notes:

Trifluorobenzoyl chloride and benzoic acid have an RT of 11.7 if not converted to the ester. This would interfere with rection monitoring.

$$*Relative\ RF = \frac{\left({}^{Analyte\ 2\ Conc.}/_{Analyte\ 2\ Peak\ Area}\right)}{\left({}^{Analyte\ 1\ Conc.}/_{Analyte\ 2\ Peak\ Area}\right)}$$

Representative Chromatogram(s) (attach additional chromatograms and spectra as needed)

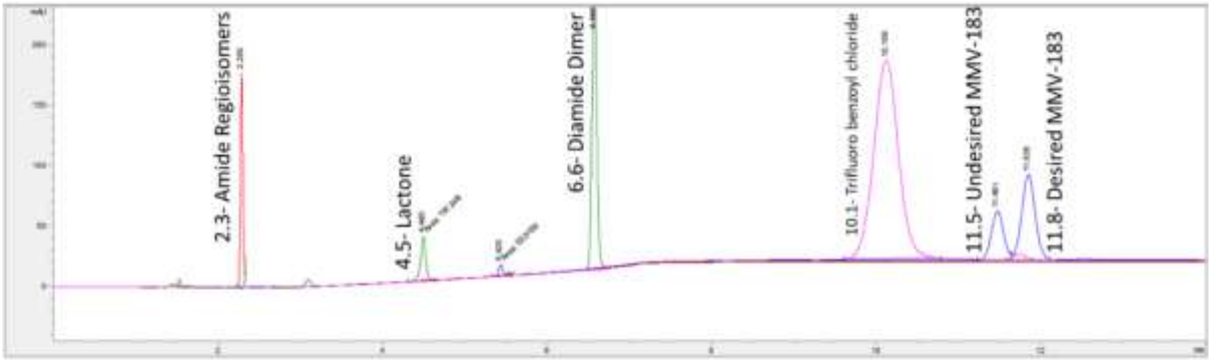

MMV-183 regioisomers:

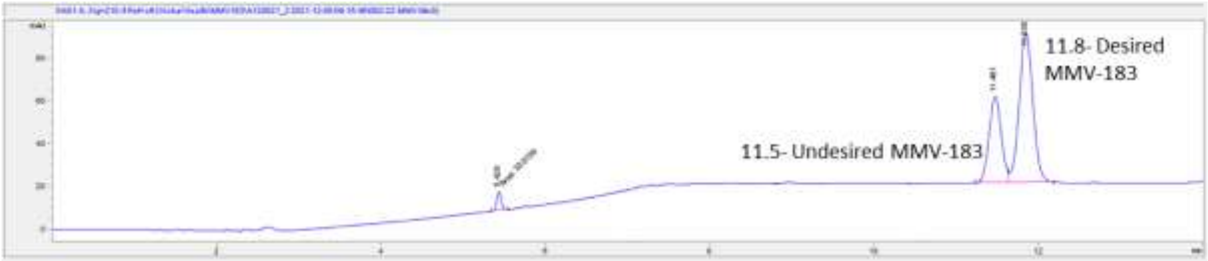

Amide regioisomers 11+12 as a inseparable mixture:

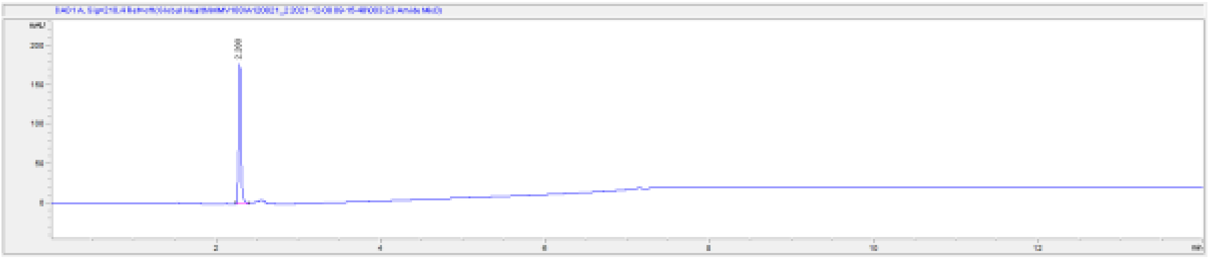

Trifluoro benzoyl chloride UV:

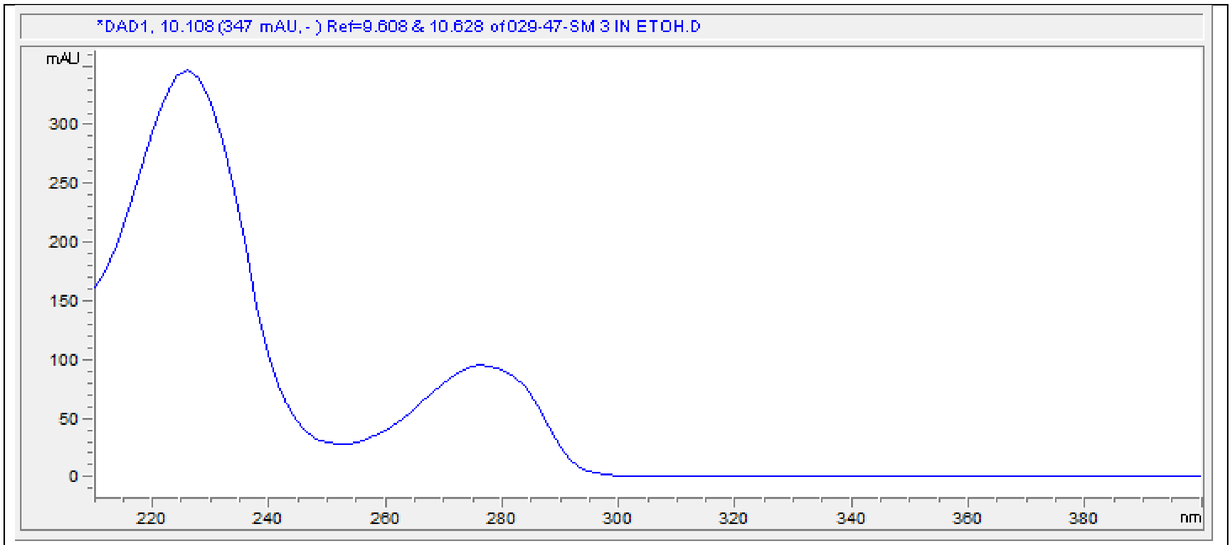

Lactone UV:

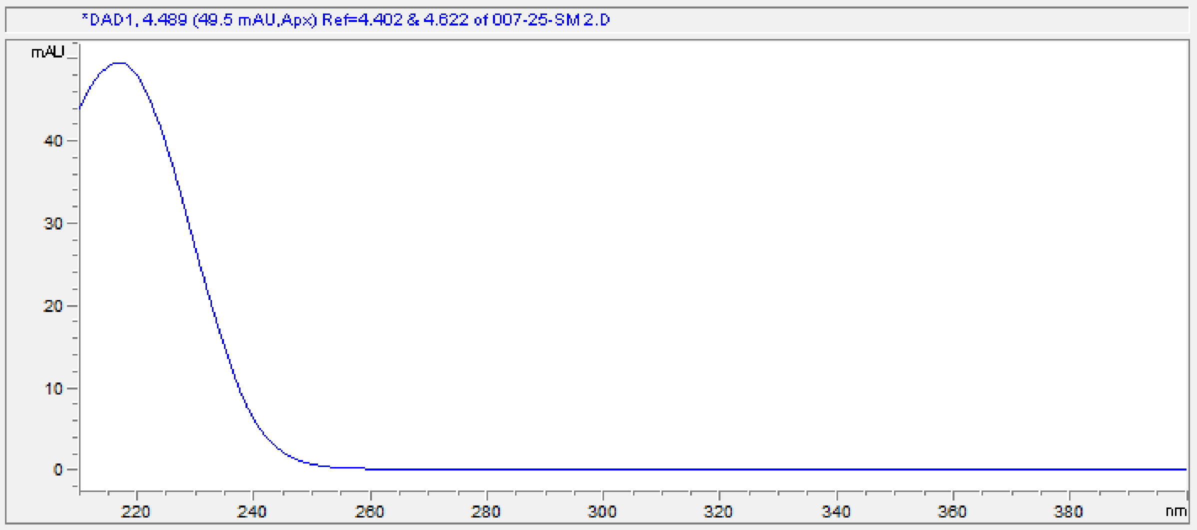

Amide regioisomers and dimer UV:

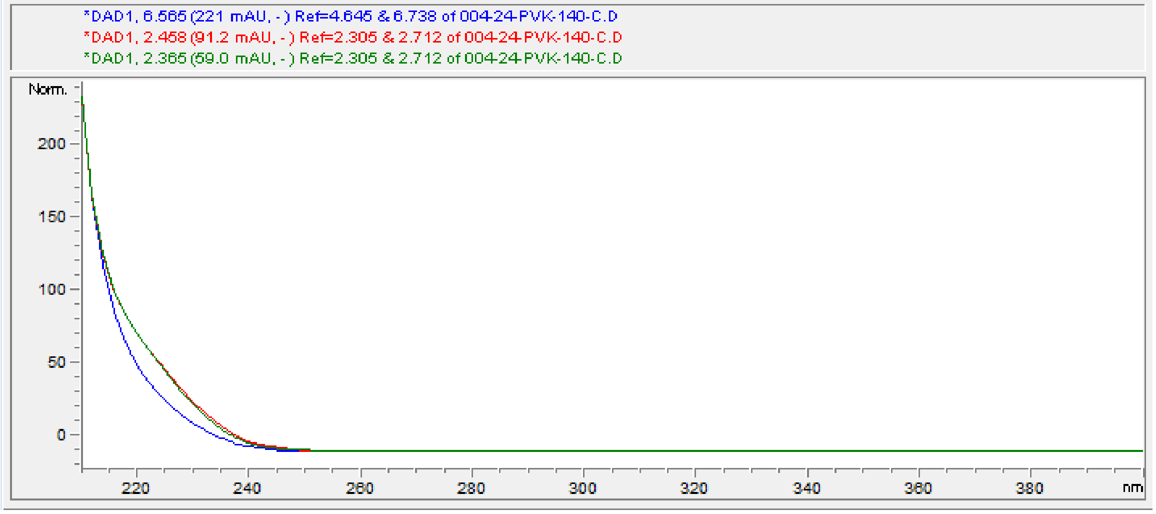

MMV-183 regioisomers UV:

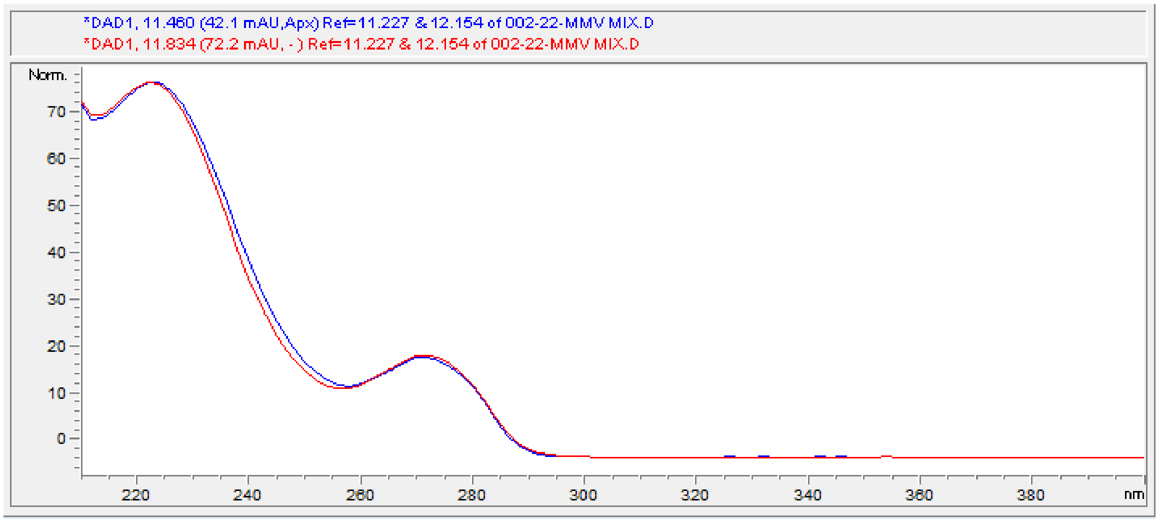

__**For comparison, the undesired isomer of MMV693183 was prepared from pure compound 12:**__

Synthesis of N-((S)-2-((R)-2,4-dihydroxy-3,3-dimethylbutanamido)propyl)-2,4,5-trifluorobenzamide (MMV693183-Undesired)

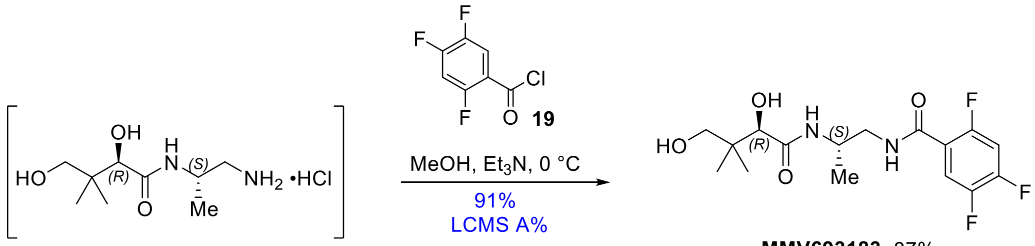

12 **MMV693183**, 87% Undesired Regioisomer

To an oven dried two-necks round bottom flask with magnetic stirrer was added HCl salt **12** (500 mg, 1.0 eq., 2.1 mmol) and dry MeOH (5.0 mL, salt **12** was completely soluble in MeOH) under nitrogen atmosphere. The mixture was cooled to 0 °C with an ice bath and Et_3N (0.43 mL, 1.5 eq., 3.1 mmol) was added. The resulting mixture was allowed to stir for 15 mins at the same temperature. To this mixture was added trifluorobenzoyl chloride **19** (0.29 mL, 1.1 eq., 2.3 mmol) dropwise 0 °C and the resulting mixture was stirred for 1 h. After completion (monitored by TLC) the reaction mixture was allowed to warm to 25 °C. The organic solvent was removed under vacuum and the resulting crude mixture was purified by column chromatography (gradient: hexanes to 1:9 EtOAc:hexanes) to afford the pure undesired regioisomer of **MMV693183-undesired** (0.66 g, 87%).) to afford the pure undesired regioisomer of **MMV693183-undesired** (0.66 g, 87%), which exhibits different ^{1}H, ^{13}C and ^{19}FNMR and melting point.

^{1}H NMR (600 MHz, CD_3OD) δ/ppm: 7.74-7.66 (m, 1H), 7.34-7.27 (m, 1H), 4.21-4.13 (m, 1H), 3.87 (s, 1H), 3.54-3.32 (m, 4H), 1.21 (d, J = 6.7 Hz, 3H), 0.87 (s, 3H), 0.86 (s, 3H).

^{13}C NMR (150 MHz, CD_3OD) δ/ppm: 175.9, 164.8, 157.0 (ddd, J = 250.0, 10.0, 2.2 Hz), 153.1 (dt, J = 254.0, 13.8 Hz), 148.1 (ddd, J = 245.0, 13.0, 3.0 Hz), 120.7 (dt, J = 16.5, 4.4 Hz), 119.7 (dd, J = 21.0, 2.5 Hz), 107.6 (dd, J = 23.6, 8.0 Hz), 77.4, 70.2, 46.5, 40.3, 21.5, 20.9, 18.3.

^{19}F NMR (564 MHz, CD_3OD) δ/ppm: -114.3 (dd, J = 17.2, 6.5 Hz, 1F), -130.7 (dd, J = 22.6, 6.3 Hz, 1F), -142.6 (dd, J = 23.1, 6.4 Hz, 1F).

HRMS (ESI) *m/z*: $[M + Na]^+$ Calculated for $C_{16}H_{21}F_3N_2O_4Na$: 385.1453; Found: 385.1462

Melting Point: 106 °C.

II. ^{1}H, ^{19}F and ^{13}C NMR Spectra of all new compounds:

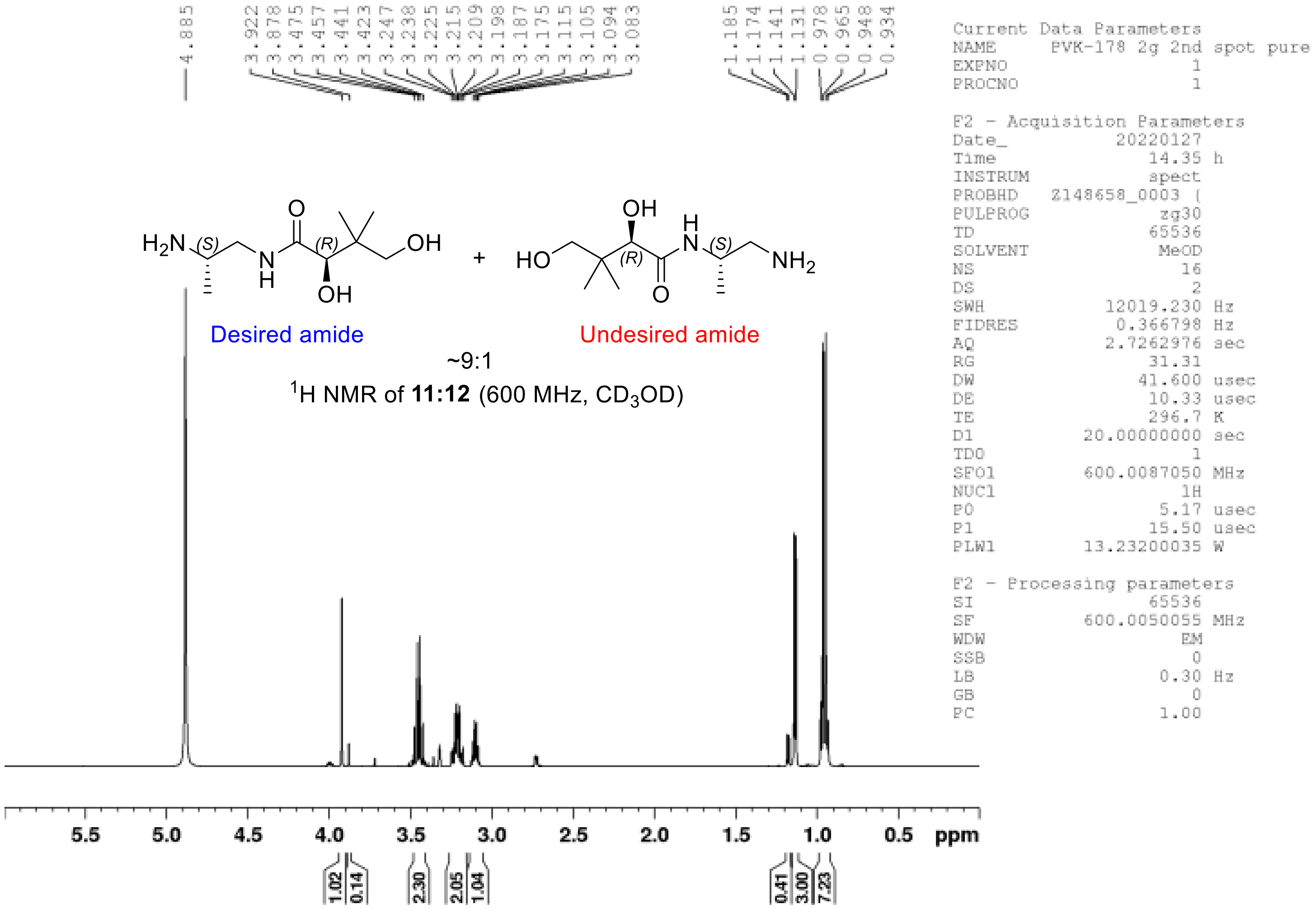

Fig S1. ^{1}HNMR of compound **11** in CD_3OD.

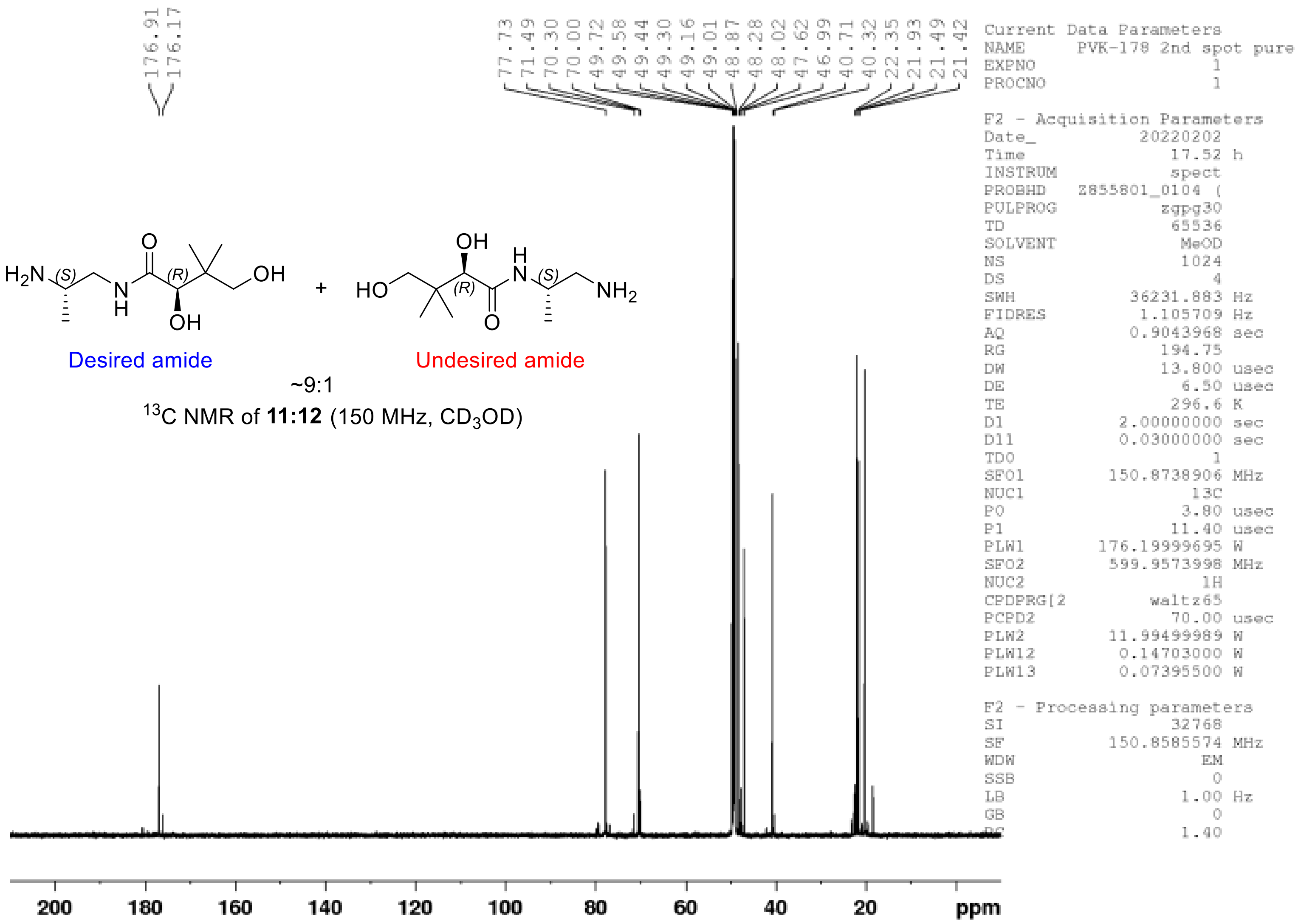

Fig S2. ^{13}CNMR of compound **11** in CD_3OD

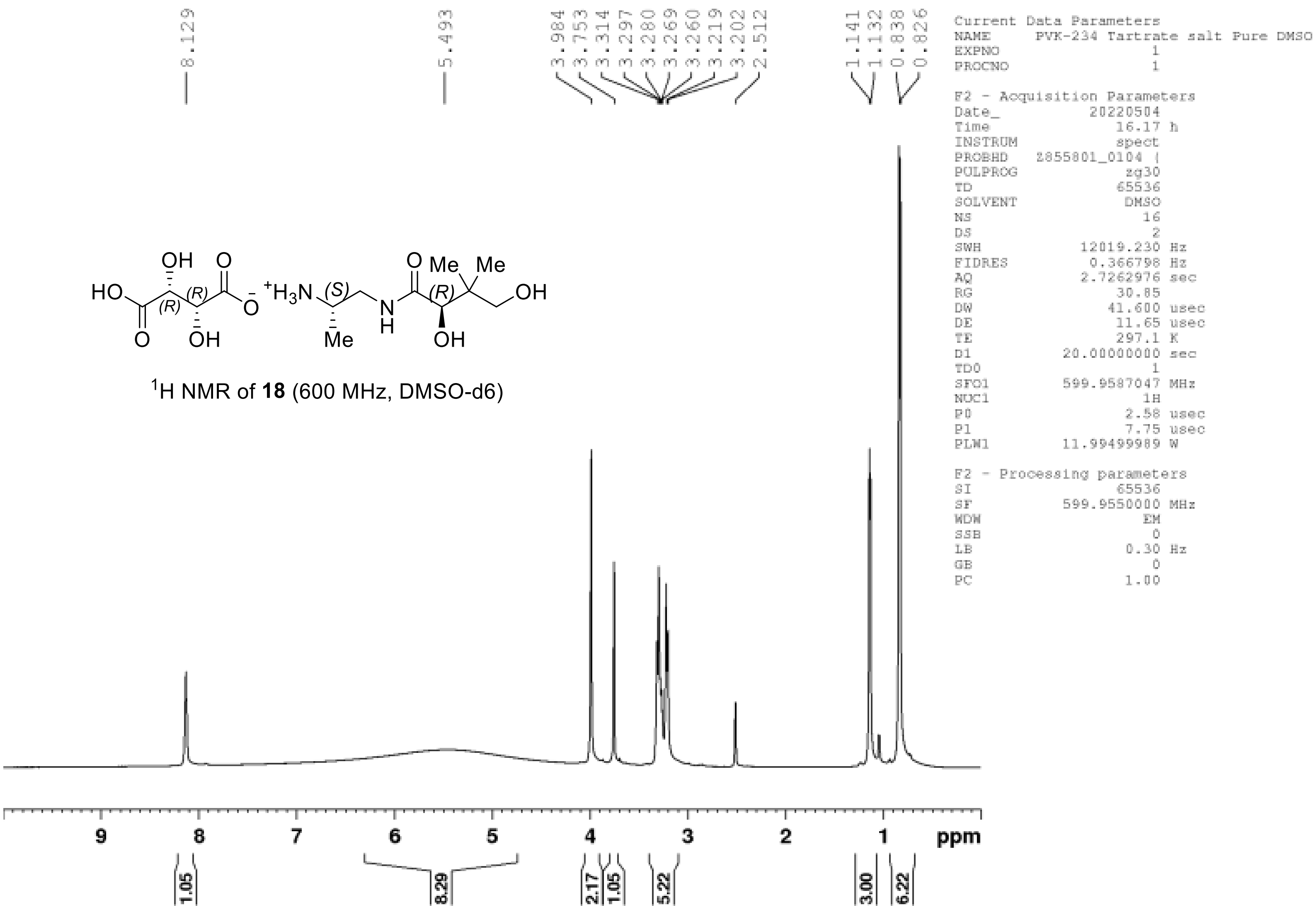

Fig S3. ^{1}HNMR of compound **18** in DMSO-d$_6$

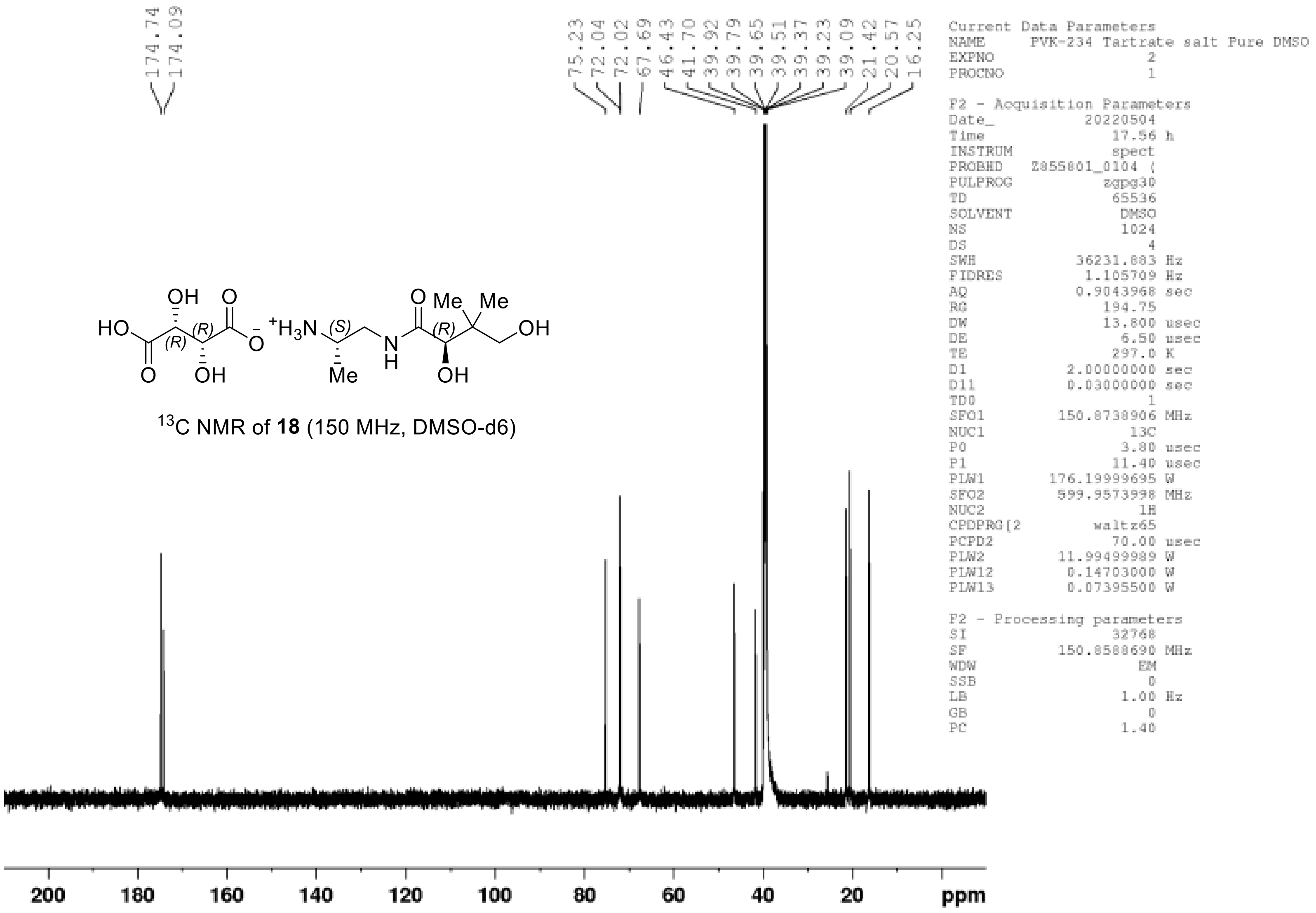

Fig S4. ^{13}CNMR of compound **18** in DMSO-d$_6$

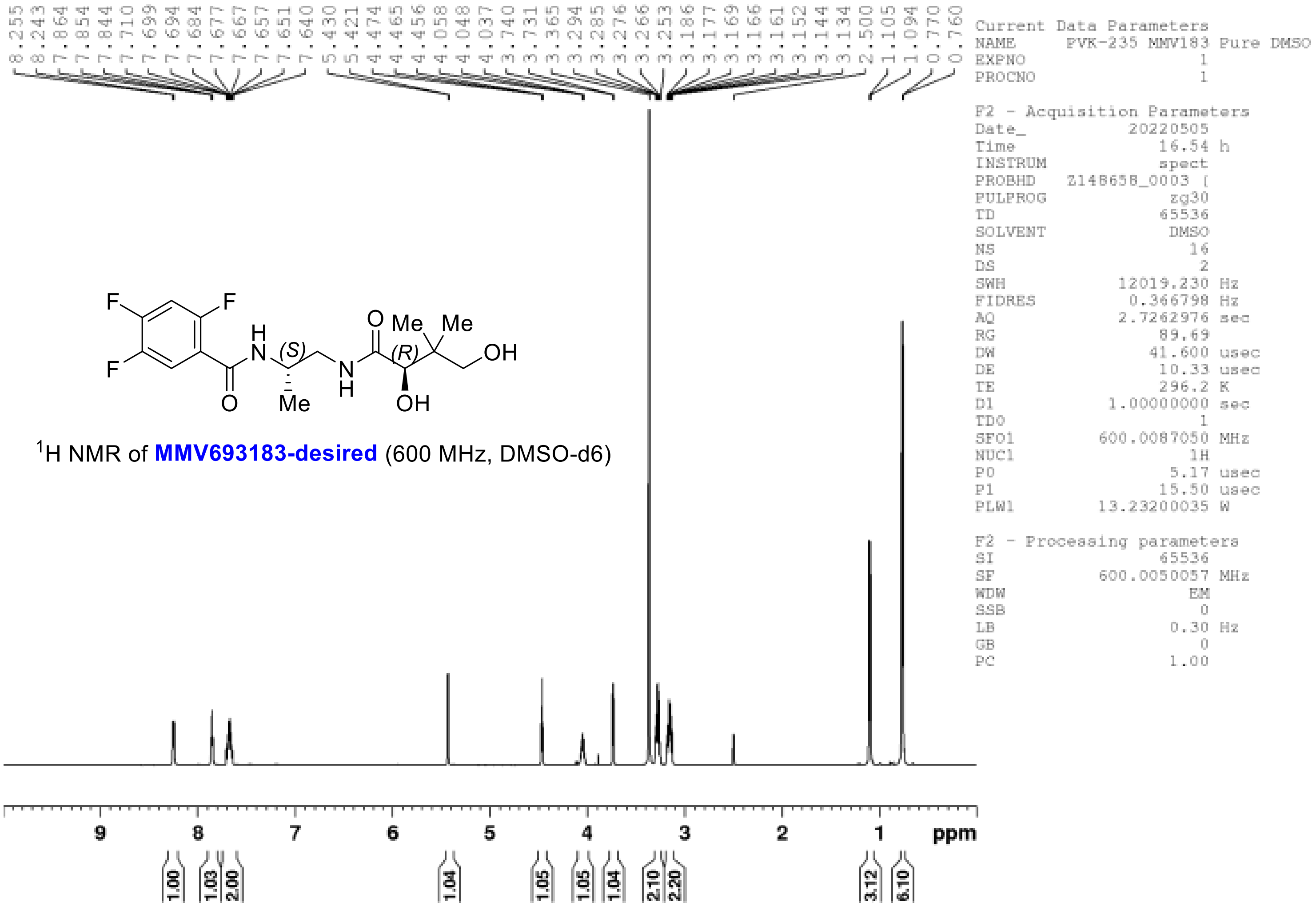

Fig S5. ^{1}HNMR data of MMV693183 (desired isomer) in DMSO-d_6

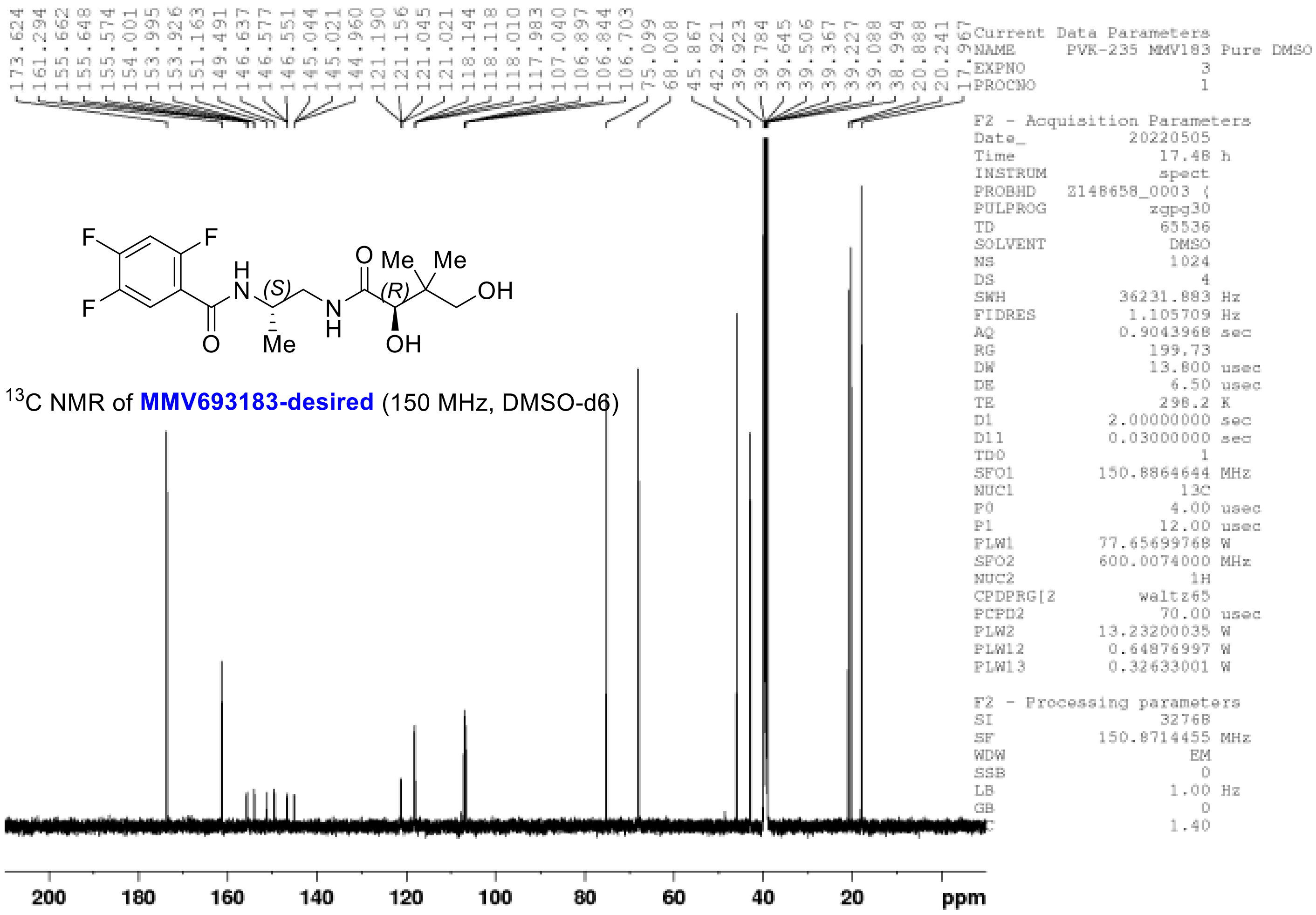

Fig S6. ^{13}CNMR data of MMV693183 (desired isomer) in DMSO-d_6

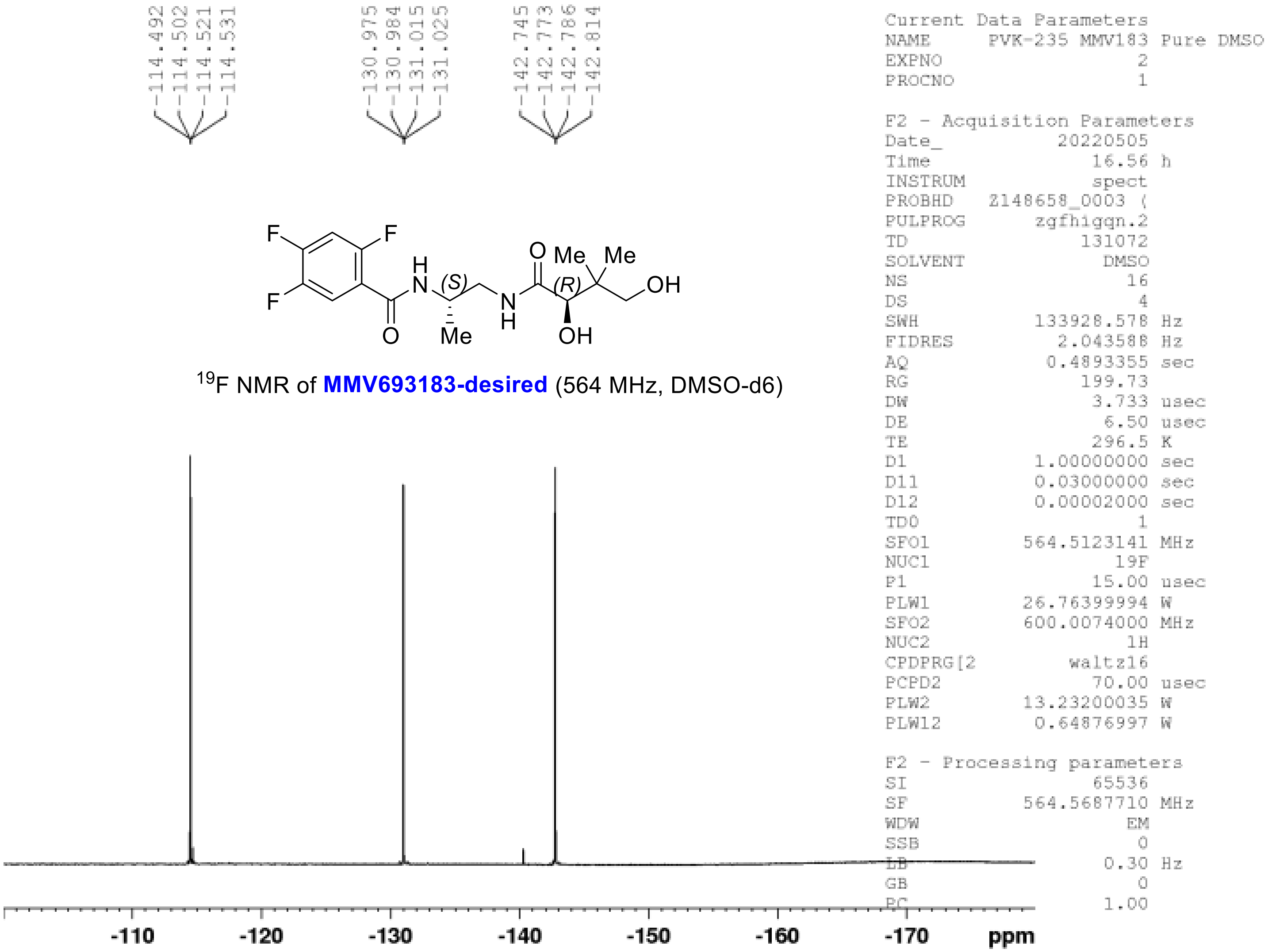

Fig S7. ^{19}FNMR data of MMV693183 (desired isomer) in DMSO-d_6

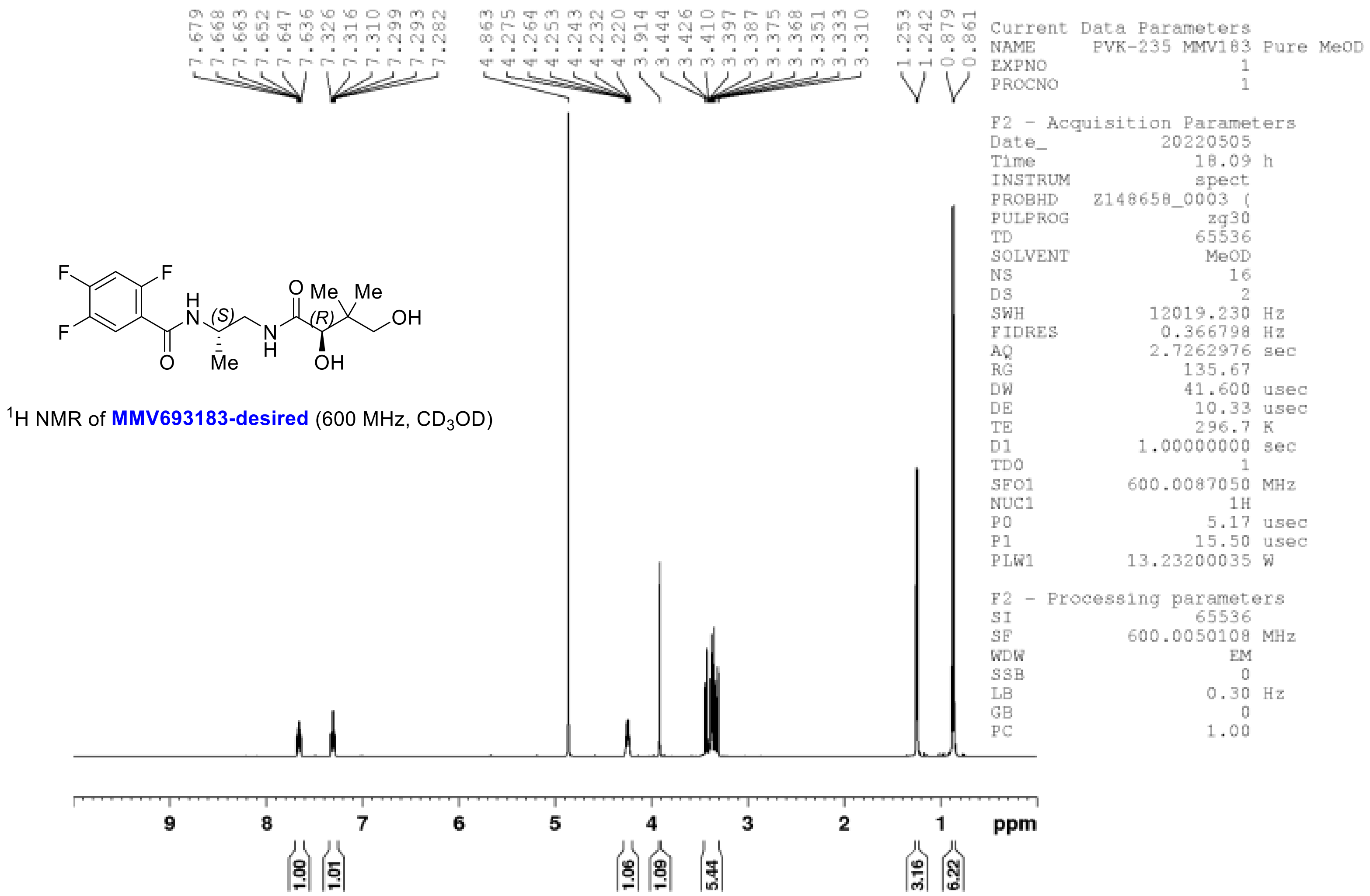

Fig S8. ^{1}HNMR data of MMV693183 (desired isomer) in CD_3OD

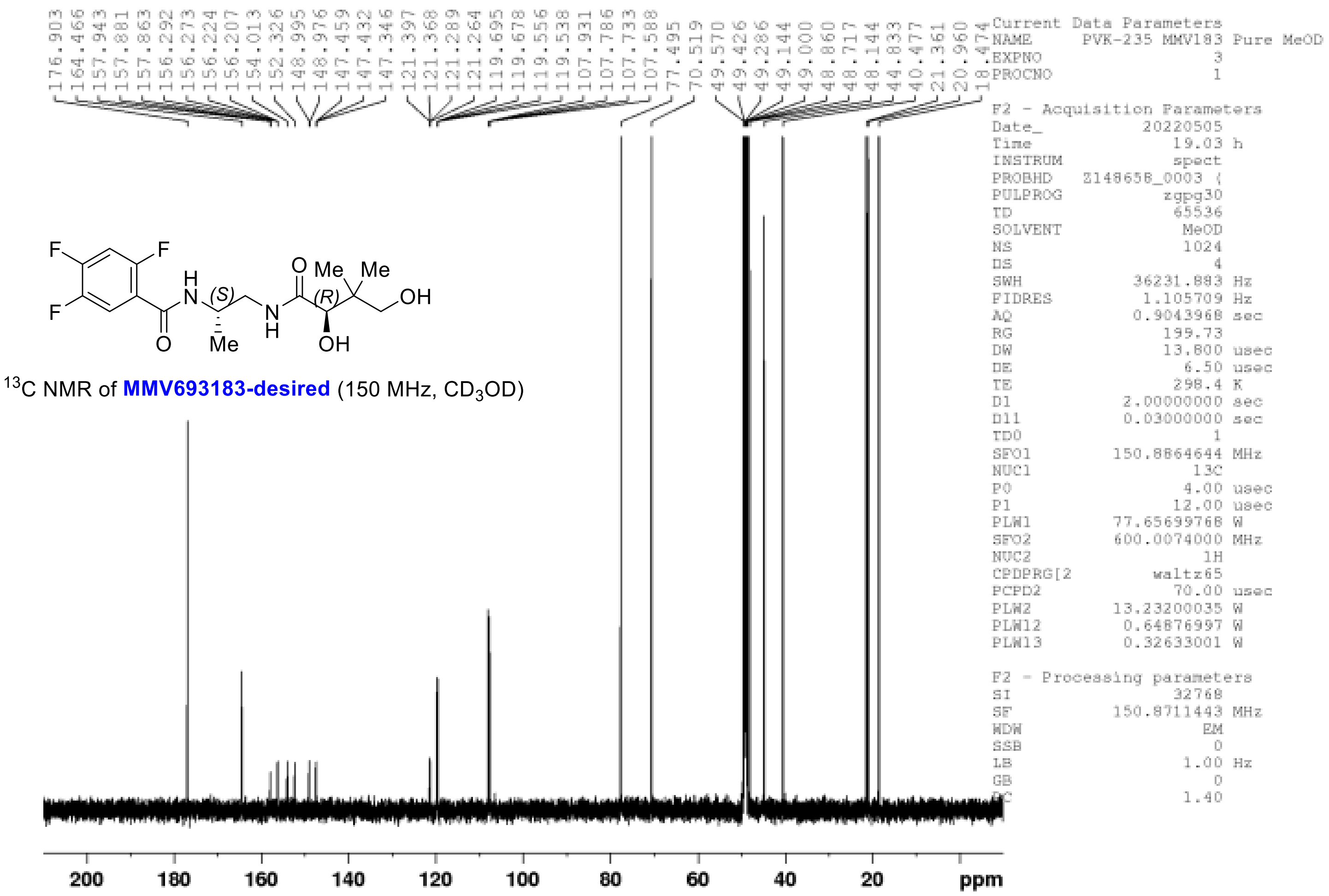

Fig S9. ^{13}CNMR data of MMV693183 (desired isomer) in CD_3OD

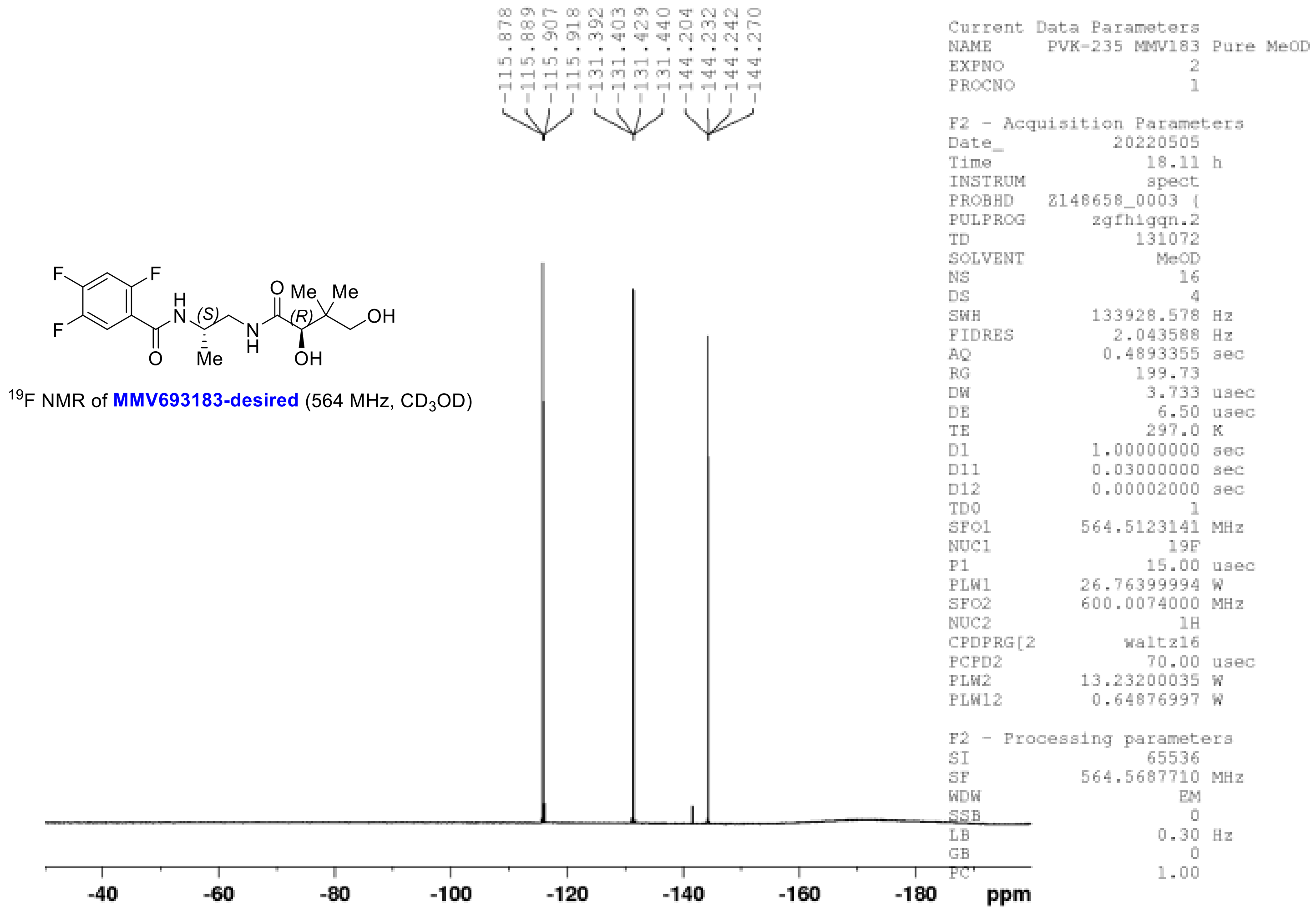

Fig S10. ^{19}FNMR data of MMV693183 (desired isomer) in CD_3OD

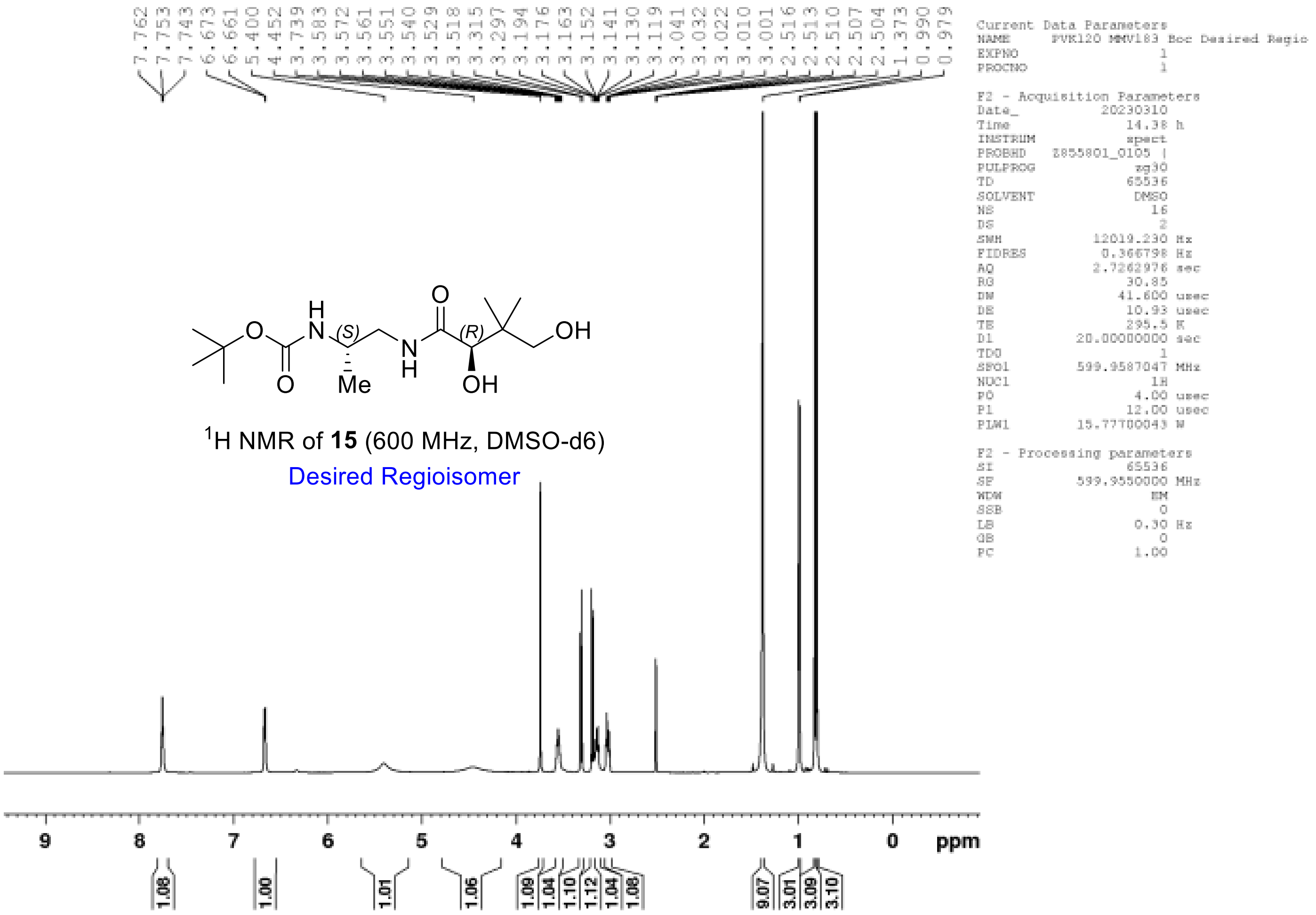

Fig S11. ^{1}HNMR data of compound **15** in DMSO-d_6

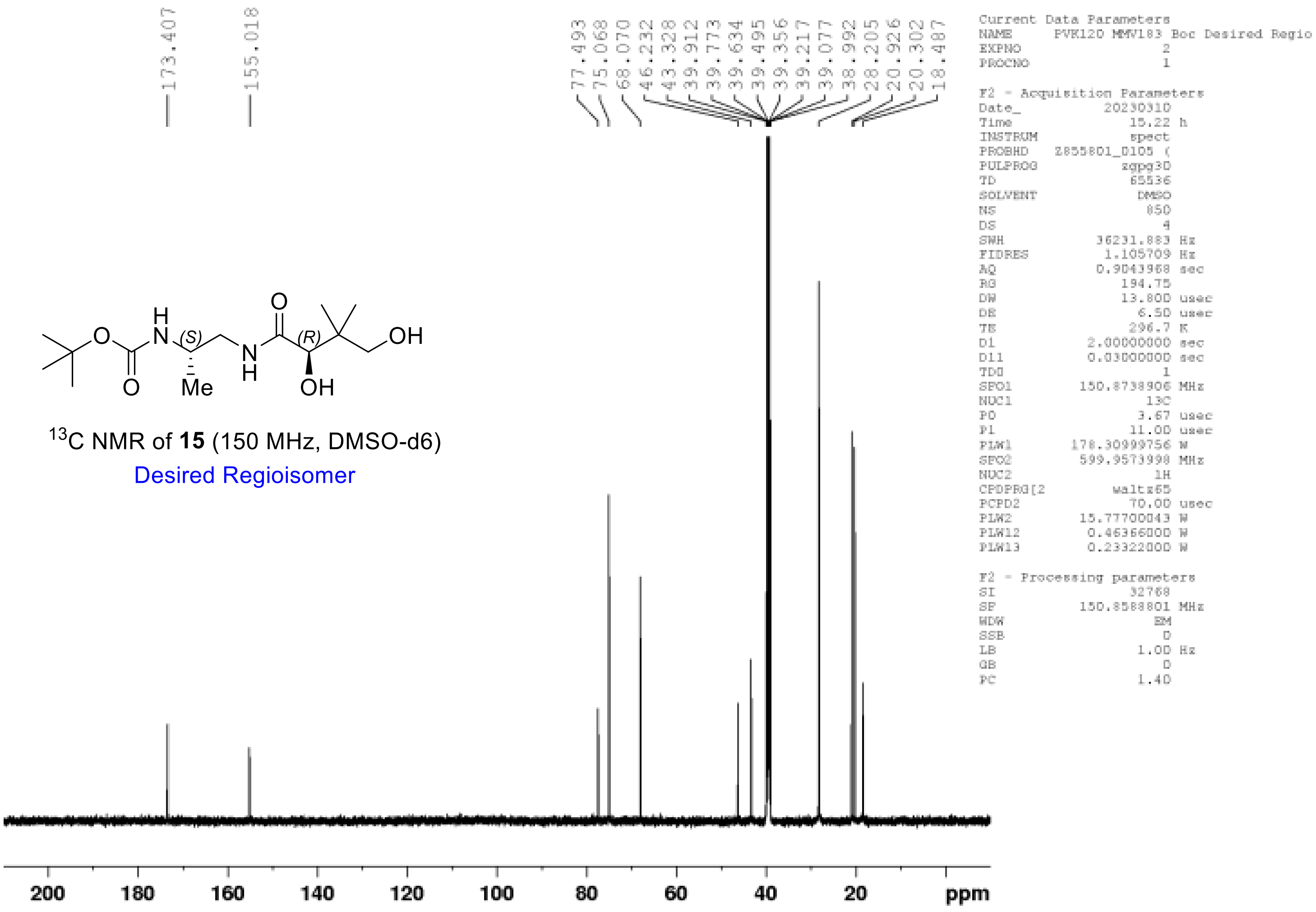

Fig S12. ^{13}CNMR data of compound **15** in DMSO-d_6

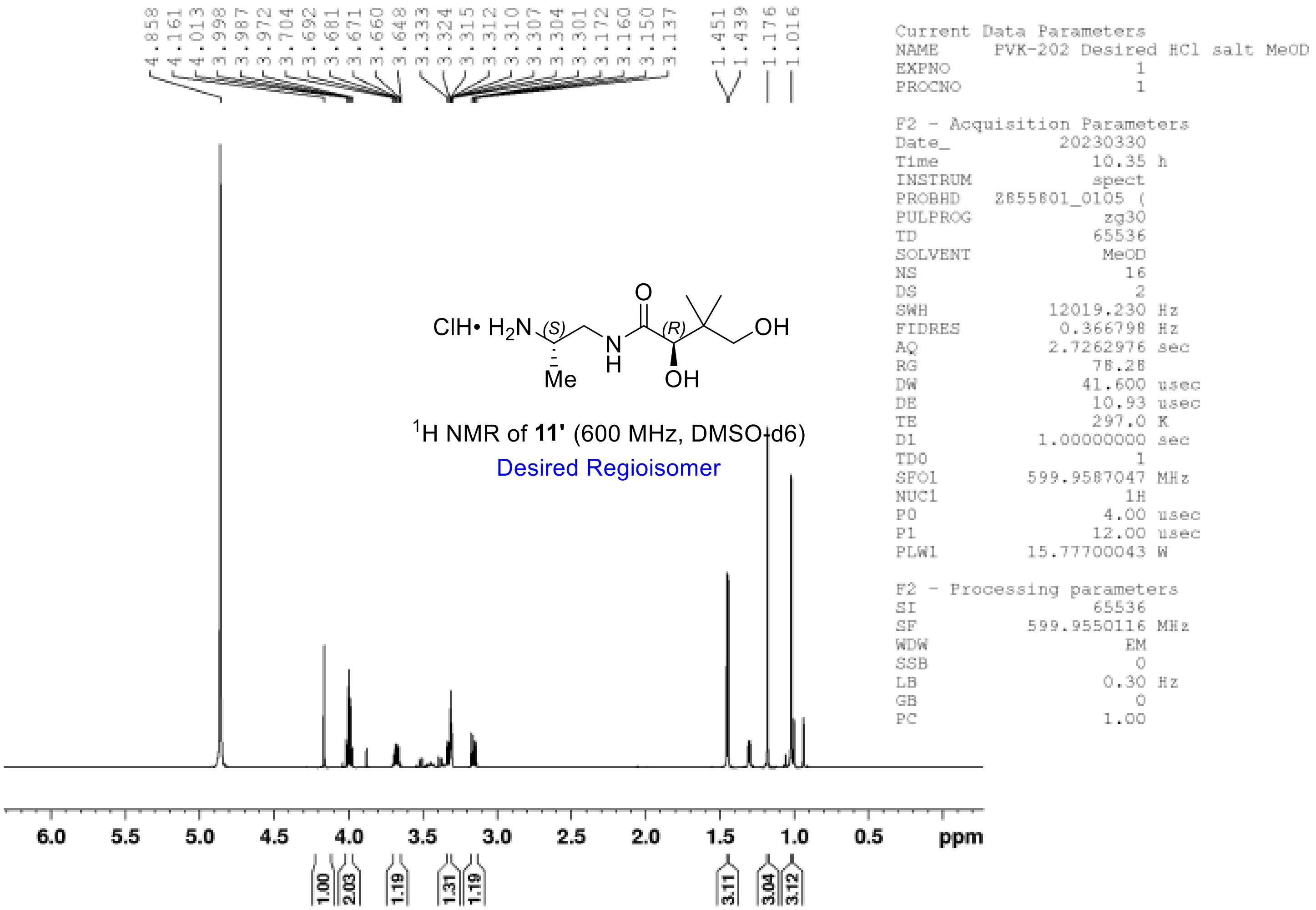

Fig S13. ^{1}HNMR data of compound **11′** in DMSO-d_6

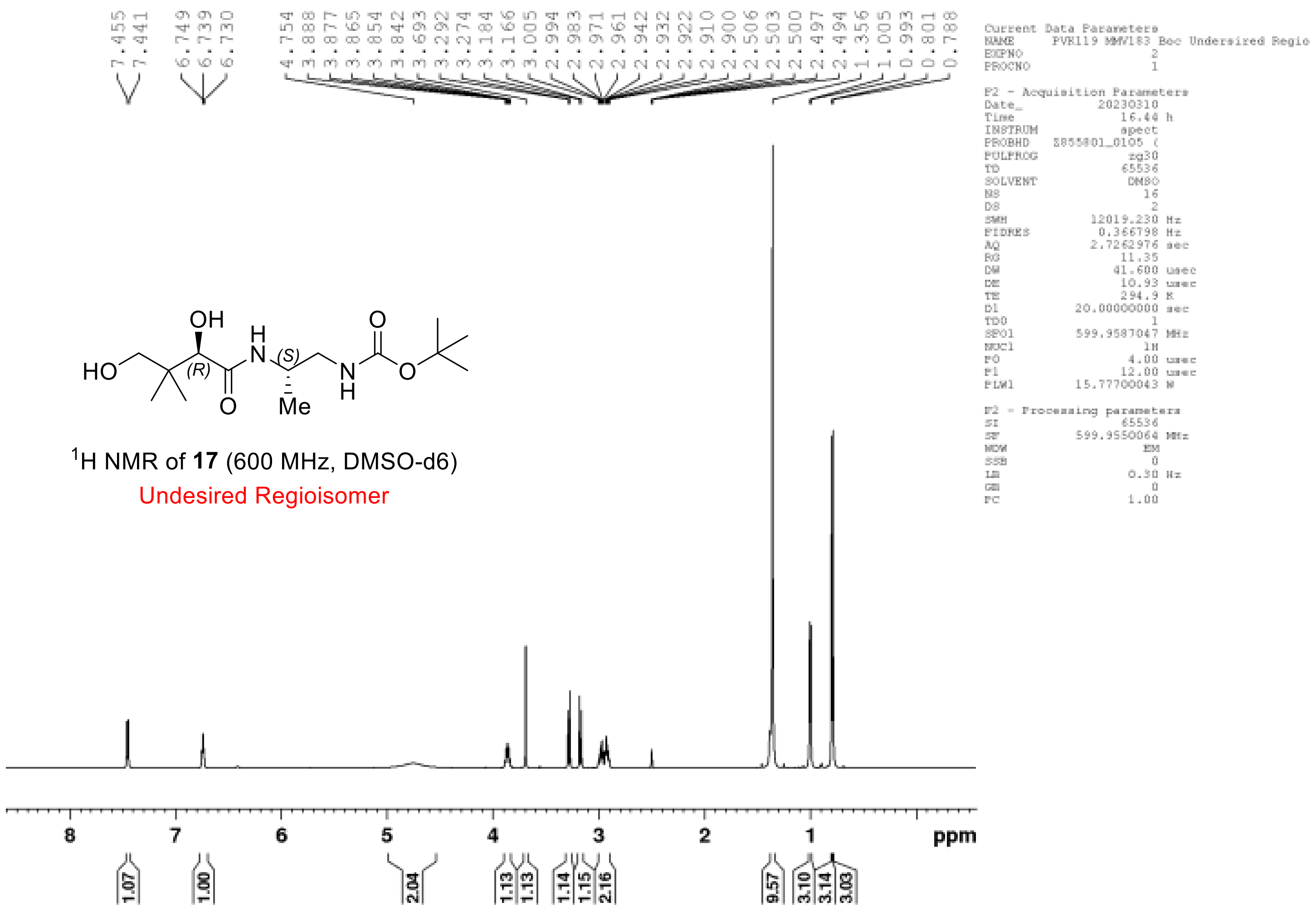

Fig S14. ^{1}HNMR data of compound **17** in DMSO-d_6

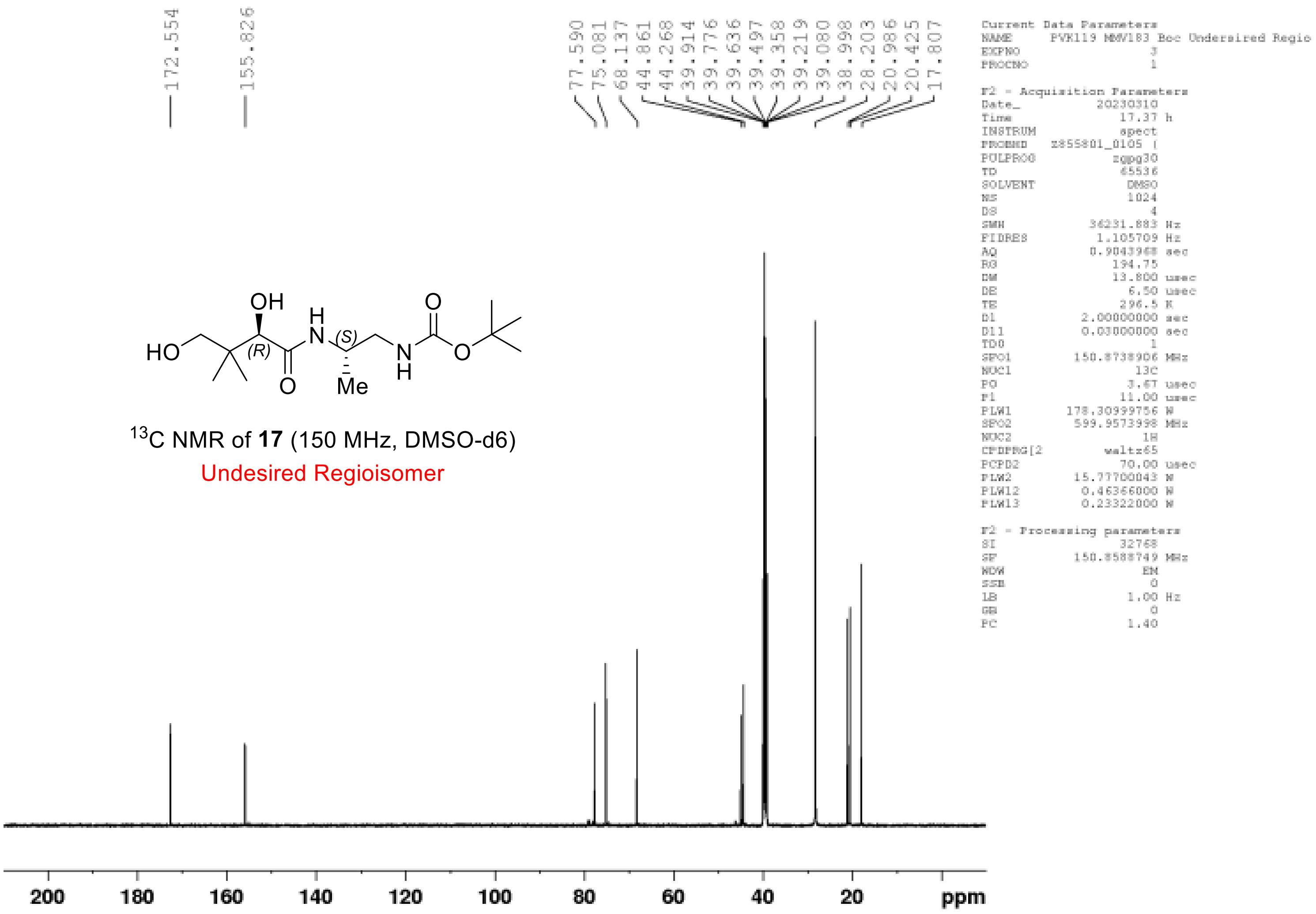

Fig S15. ^{13}CNMR data of compound **17** in DMSO-d_6

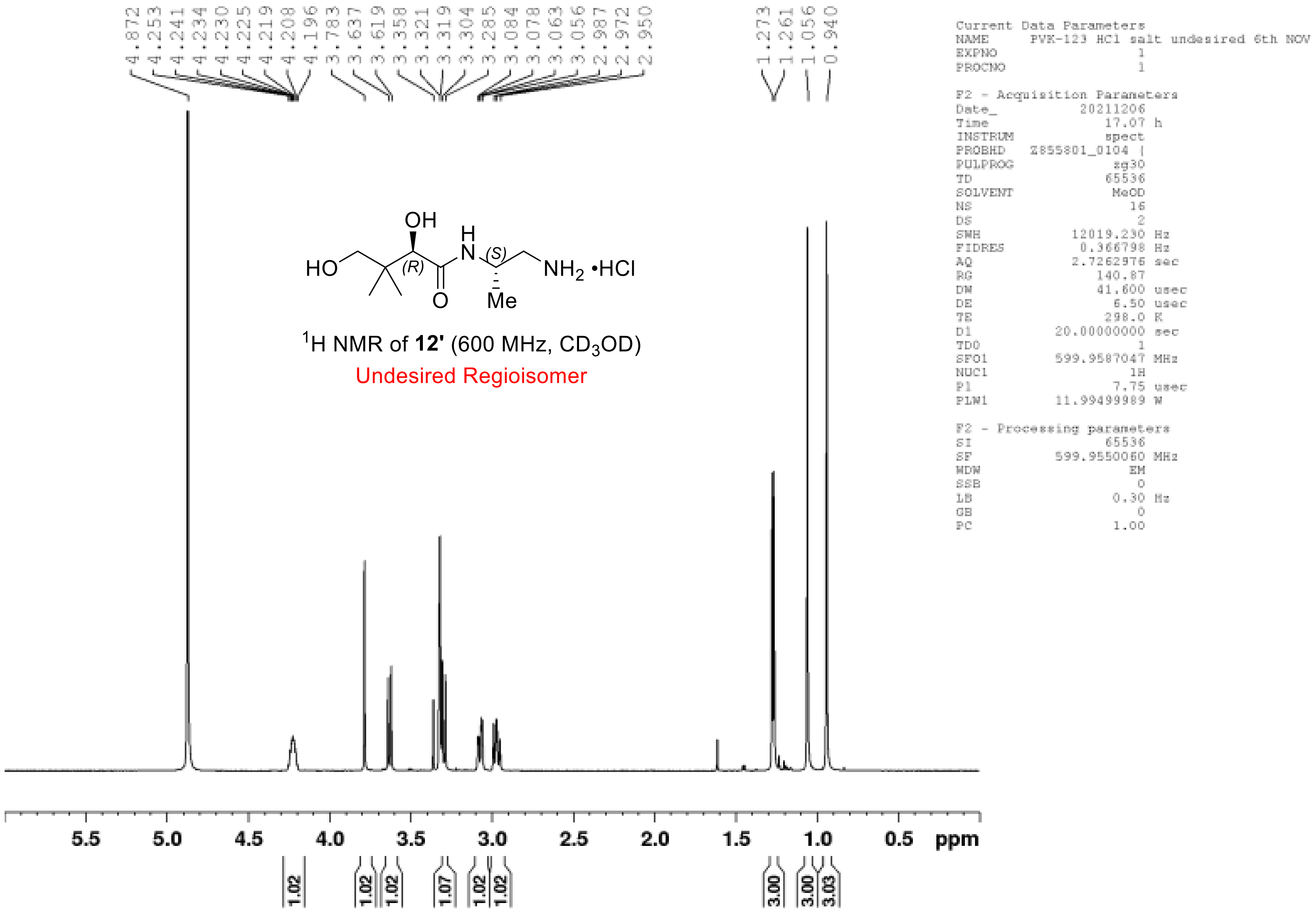

Fig S16. ^{1}HNMR data of compound **12′** in CD_3OD

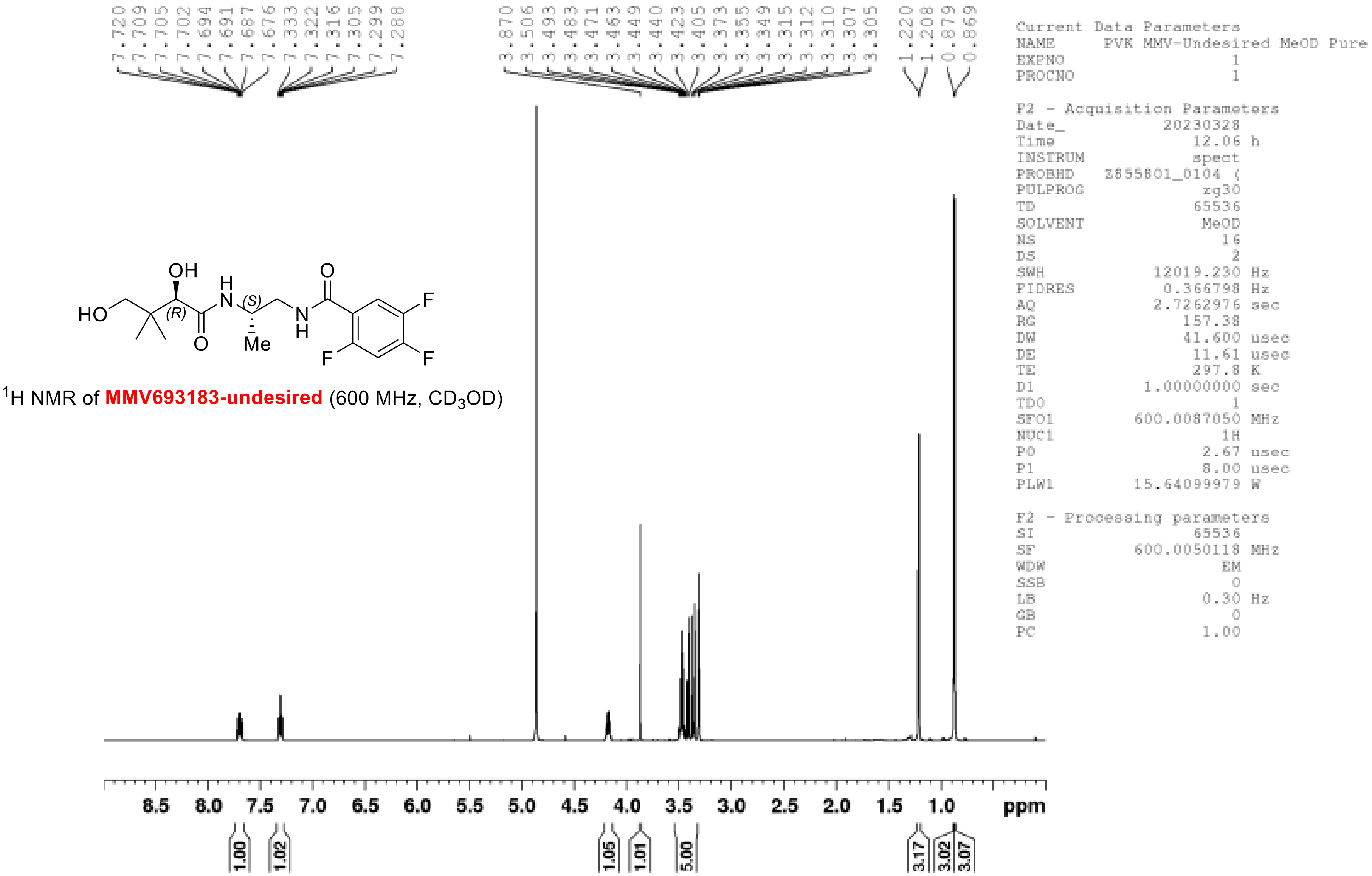

Fig S17. ^{1}HNMR data of MMV693183-undesired isomer in CD_3OD

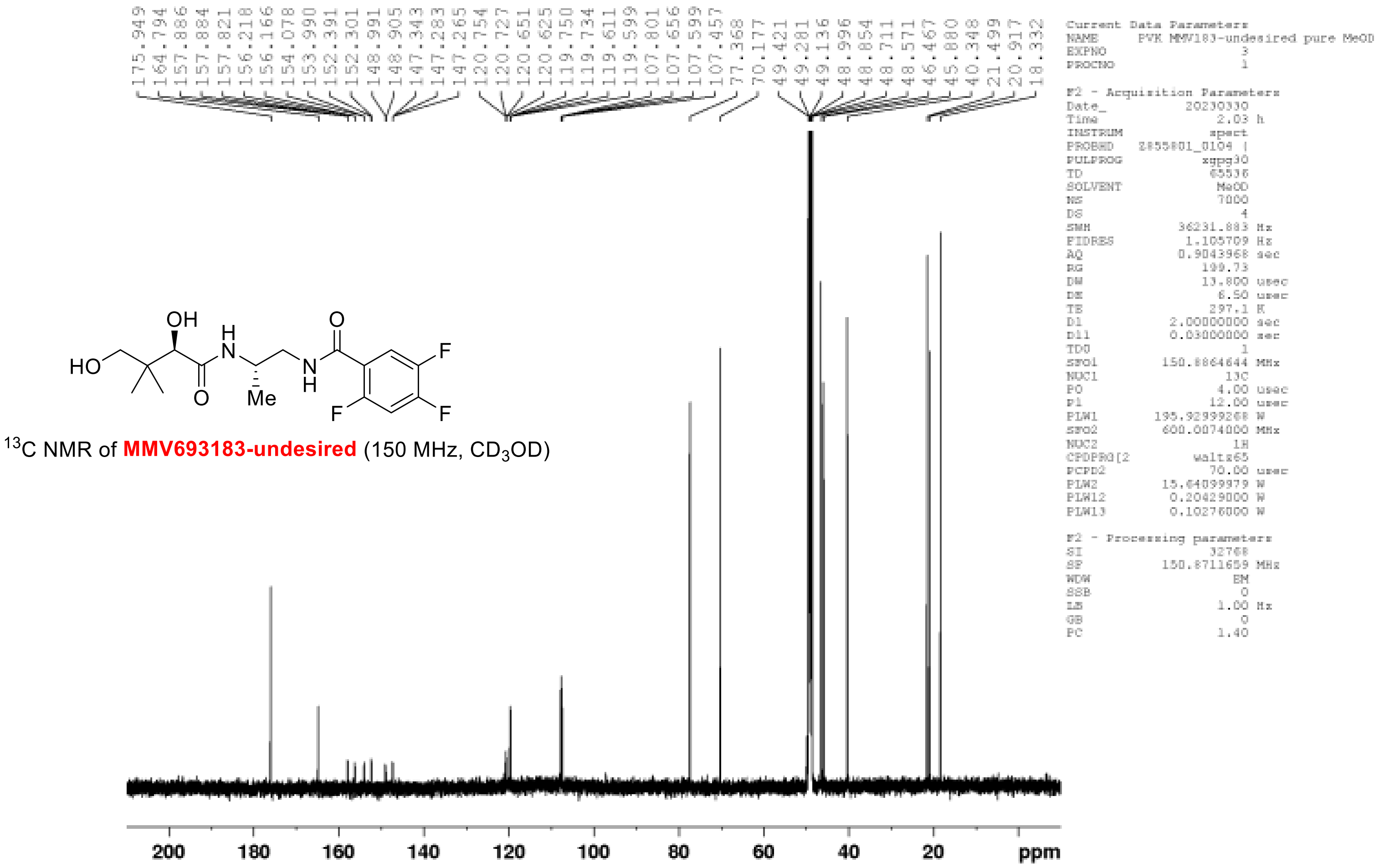

Fig S18. ^{13}CNMR data of MMV693183-undesired isomer in CD_3OD

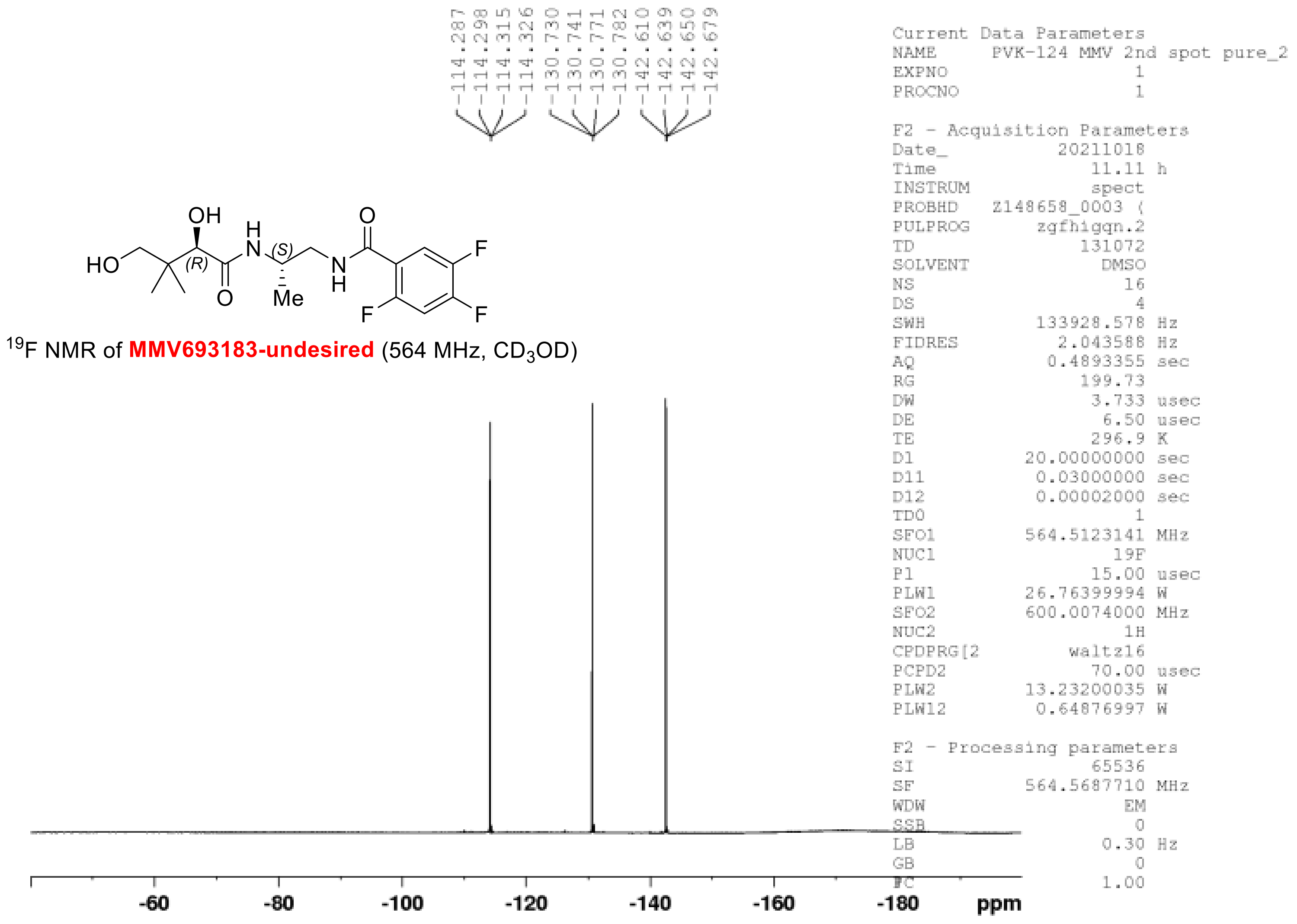

Fig S19. ^{19}FNMR data of MMV693183-undesired isomer in CD_3OD

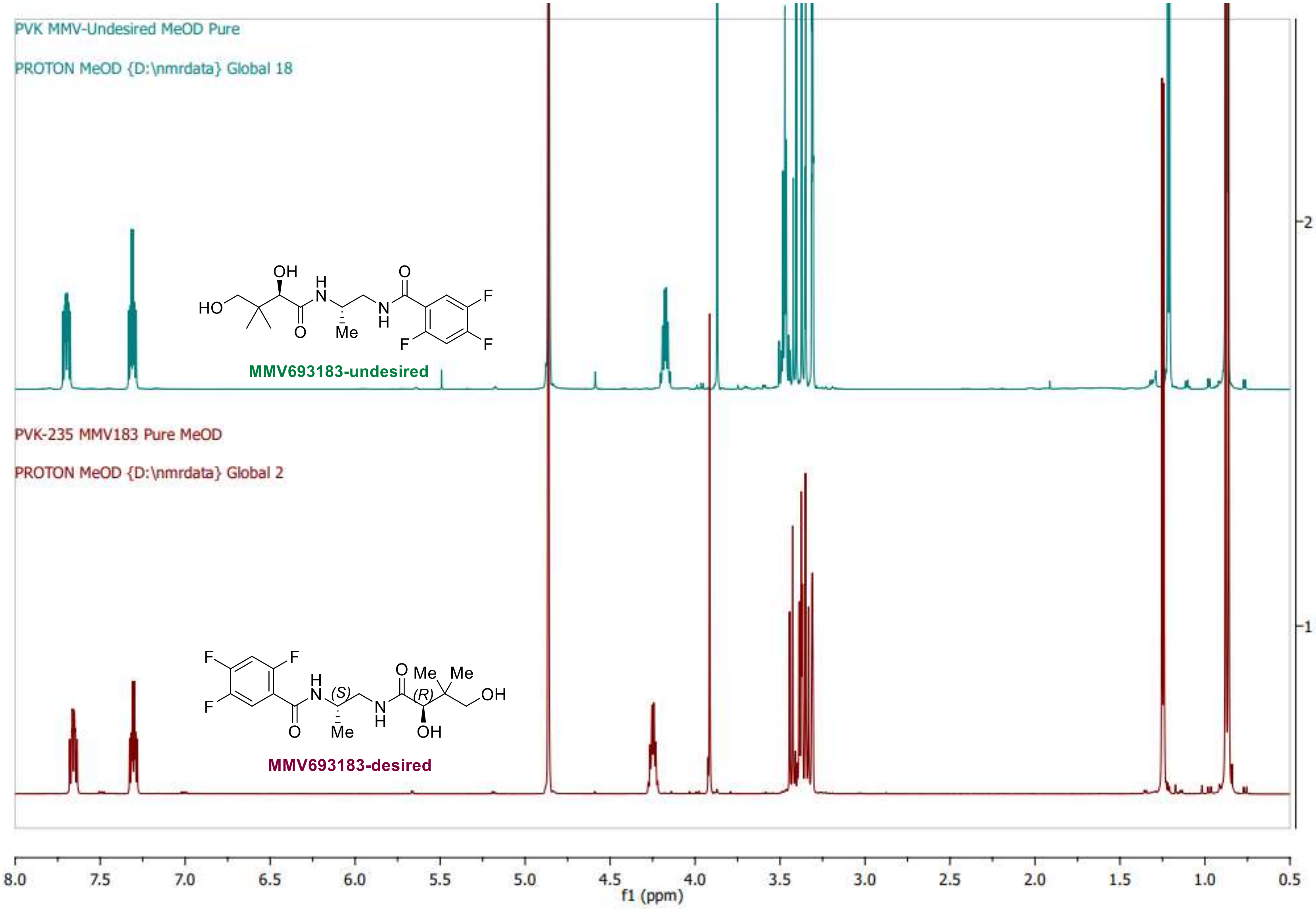

Fig S20. ^{1}HNMR data comparison of MMV693183 (desired and undesired regisomers) in CD_3OD

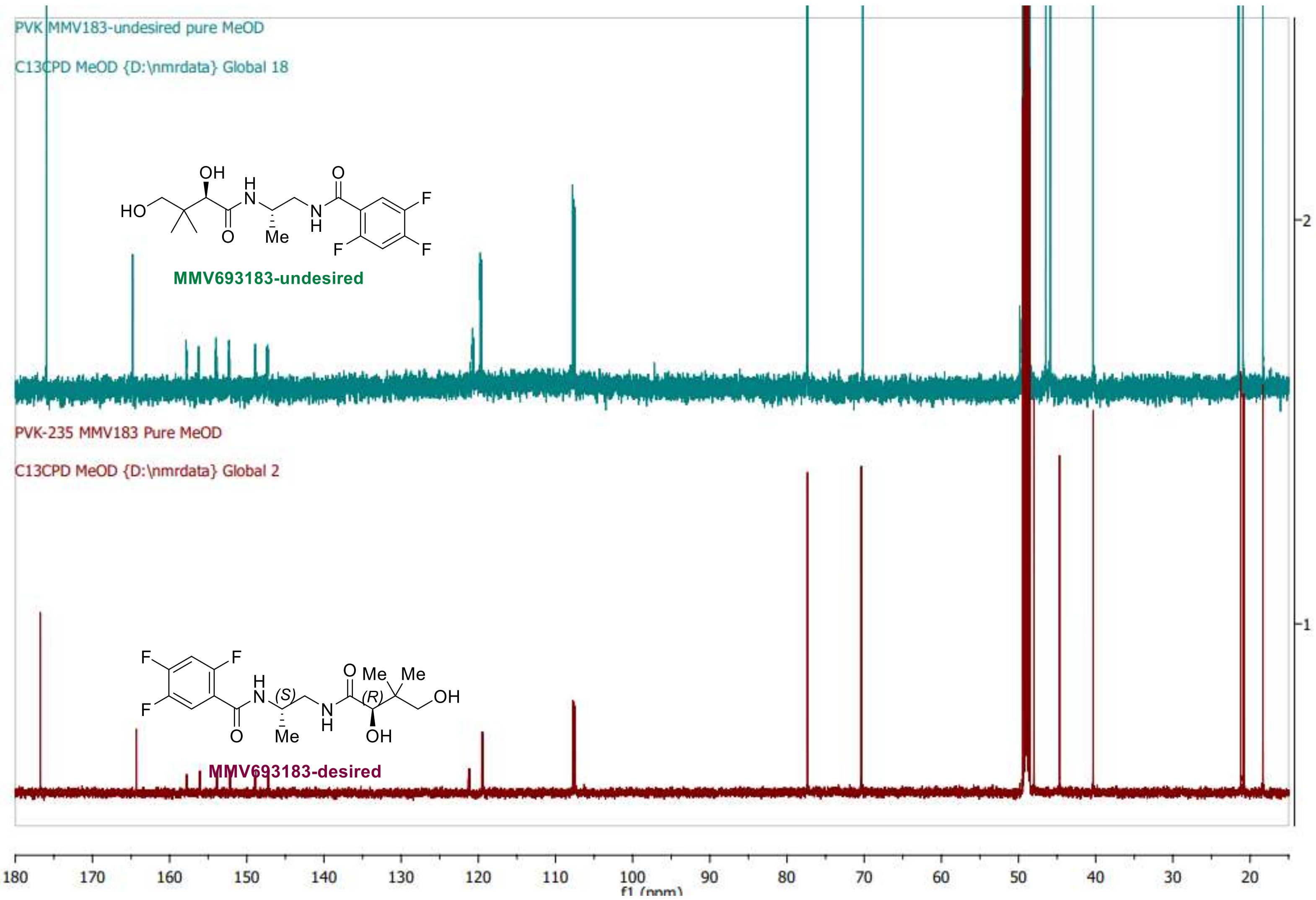

Fig S21. ^{13}CNMR data comparison of MMV693183 (desired and undesired regisomers) in CD_3OD
